# Embeddability of centrosymmetric matrices capturing the double-helix structure in natural and synthetic DNA

**DOI:** 10.1007/s00285-023-01895-8

**Published:** 2023-04-05

**Authors:** Muhammad Ardiyansyah, Dimitra Kosta, Jordi Roca-Lacostena

**Affiliations:** 1 Department of Mathematics and Systems Analysis, Aalto University, Espoo, Finland; 2 School of Mathematics, University of Edinburgh and Maxwell Institute for Mathematical Sciences, Edinburgh, UK; 3 Universitat Politécnica de Catalunya, Barcelona, Catalunya, Spain

**Keywords:** Evolutionary model, Embedding problem, Markov matrix, Centrosymmetric matrix, 60J10, 60J27, 15B51, 15A16, 92D15

## Abstract

In this paper, we discuss the embedding problem for centrosymmetric matrices, which are higher order generalizations of the matrices occurring in strand symmetric models. These models capture the substitution symmetries arising from the double helix structure of the DNA. Deciding whether a transition matrix is embeddable or not enables us to know if the observed substitution probabilities are consistent with a homogeneous continuous time substitution model, such as the Kimura models, the Jukes-Cantor model or the general time-reversible model. On the other hand, the generalization to higher order matrices is motivated by the setting of synthetic biology, which works with different sizes of genetic alphabets.

## Introduction

Phylogenetics is the study of evolutionary relationships among biological entities, also known as taxa, that aims to infer the evolutionary history among them. In order to model evolution, we consider a directed acyclic graph, called a phylogenetic tree, depicting the evolutionary relationships amongst a selected set of taxa. Phylogenetic trees consist of vertices and edges. Vertices represent taxa, while edges between vertices represent the evolutionary processes between the taxa.

In order to describe the real evolutionary process along an edge of a phylogenetic tree, one often assumes that the evolutionary data occurred following a Markov process. A Markov process is a random process in which the future is independent of the past, given the present. Under this Markov process, transitions between *n* states given by conditional probabilities are presented in a $n\times n$ Markov matrix *M*, that is a square matrix whose entries are nonnegative and rows sum to one. A well-known problem in probability theory is the so-called embedding problem which was initially posed by Elfving (Elfving 1937). The embedding problem asks whether given a Markov matrix *M*, one can find a real square matrix *Q* with rows summing to zero and non-negative off-diagonal entries, such that $M=\exp (Q)$. The matrix *Q* is called a Markov generator.

In the complex setting, the embedding problem is completely solved by Higham (2008); a complex matrix *A* is embeddable if and only if *A* is invertible. However, as our motivation arises from molecular models of evolution we are interested in the embedding problem over the real numbers, so from now on we will denote by *M* a real Markov matrix. It was shown by Kingman (1962) that if an $n \times n$ real Markov matrix *M* is embeddable, then the matrix *M* has $\det {M}>0$. Moreover, in the same work by Kingman it was shown that $\det {M}>0$ is a necessary and sufficient condition for a $2\times 2$ Markov matrix *M* to be embeddable. For $3\times 3$ Markov matrices a complete solution of the embedding problem is provided in a series of papers (James 1973; Johansen 1974; Carette 1995; Chen and Chen 2011), where the characterisation of embeddable matrices depends on the Jordan decomposition of the Markov matrix. For $4 \times 4$ Markov matrices the embedding problem is completely settled in a series of papers (Casanellas et al. 2020a, 2023; Roca-Lacostena and Fernández-Sánchez 2018a), where similarly to the $3\times 3$ case the full characterisation of embeddable matrices is distinguished into cases depending on the Jordan form of the Markov matrices.

For the general case of $n \times n$ Markov matrices, there are several results; some presenting necessary conditions (Elfving 1937; Kingman 1962; Runnenberg 1962), while others sufficient conditions (James 1973; Fuglede 1988; Goodman 1970; Davies et al. 2010) for embeddability of Markov matrices. Moreover, the embedding problem has been solved for special $n\times n$ matrices with a biological interest such as equal-input and circulant matrices (Baake and Sumner 2020), group-based models (Ardiyansyah et al. 2021) and time-reversible models (Jia 2016). Despite the fact that there is no theoretical explicit solution for the embeddability of general $n\times n$ Markov matrices, there are results (Casanellas et al. 2023) that enable us to decide whether a $n\times n$ Markov matrix with distinct eigenvalues is embeddable or not. This is achieved by providing an algorithm that outputs all Markov generators of such a Markov matrix (Casanellas et al. 2023; Roca-Lacostena 2021).

In this paper, we focus on the embedding problem for $n \times n$ matrices that are symmetric about their center and are called centrosymmetric matrices (see Definition 2). We also study a variation of the famous embedding problem called model embeddability, where apart from the requirement that the Markov matrix is the matrix exponential of a rate matrix, we additionally ask that the rate matrix follows the model structure. For instance, for centrosymmetric matrices, model embeddability means that the rate matrix is also centrosymmetric.

The motivation for studying centrosymmetric matrices comes from evolutionary biology, as the most general nucleotide substitution model when considering both DNA strands admits any $n\times n$ centrosymmetric Markov matrix as a transition matrix, where *n* is the even number of nucleotides. For instance, by considering the four natural nucleotides A-T, C-G we arrive at the strand symmetric Markov model, a well-known phylogenetic model whose substitution probabilities reflect the symmetry arising from the complementarity between the two strands that the DNA is composed of (see (Casanellas and Sullivant 2005)). In particular, a strand symmetric model for DNA must have the following equalities of probabilities in the root distribution:

1.1 $$\begin{aligned} \pi _{\texttt {A}} = \pi _{\texttt {T}} \text { and } \pi _{\texttt {C}} = \pi _{\texttt {G}} \end{aligned}$$

and the following equalities of probabilities in the transition matrices $(\theta _{ij})$

$$\begin{aligned} \theta _{\texttt {A} \texttt {A}} = \theta _{\texttt {T} \texttt {T}}, \theta _{\texttt {A} \texttt {C}} = \theta _{\texttt {T} \texttt {G}}, \theta _{\texttt {A} \texttt {G}} = \theta _{\texttt {T} \texttt {C}}, \theta _{\texttt {A} \texttt {T}} = \theta _{\texttt {T} \texttt {A}},\\ \theta _{\texttt {C} \texttt {A}} = \theta _{\texttt {G} \texttt {T}}, \theta _{\texttt {C} \texttt {C}} = \theta _{\texttt {G} \texttt {G}}, \theta _{\texttt {C} \texttt {G}} = \theta _{\texttt {G} \texttt {C}}, \theta _{\texttt {C} \texttt {T}} = \theta _{\texttt {G} \texttt {A}}. \end{aligned}$$

Therefore, the corresponding transition matrices of this model are $4\times 4$ centrosymmetric matrices, usually called strand symmetric Markov matrices in this context. In this article, we will use the terminology $4\times 4$ centrosymmetric Markov matrix and strand symmetric Markov matrix interchangeably. In the strand symmetric model there are less restrictions on the way genes mutate from ancestor to child compared to other widely known molecular models of evolution. In fact, special cases of the strand symmetric model are the group-based phylogenetic models such as the Jukes-Cantor (JC) model, the Kimura 2-parameter (K2P) and Kimura 3-parameter (K3P) models. The algebraic structure of strand symmetric models was initially studied in (Casanellas and Sullivant 2005), where it was argued that strand symmetric models capture more biologically meaningful features of real DNA sequences than the commonly used group-based models, as for instance, in any group-based model, the stationary distribution of bases for a single species is always the uniform distribution, while computational evidence in (Yap and Pachter 2004) suggests that the stationary distribution of bases for a single species is rarely uniform, but must always satisfy the symmetries (1.1) arising from nucleotide complementarity, as assumed by the strand symmetric model.

In this article, we also explore higher order centrosymmetric matrices for which $n > 4$, which is justified by the use of synthetic nucleotides. One of main goals of synthetic biology is to expand the genetic alphabet to include an unnatural or synthetic base pair. The more letters in a genetic system could possibly lead to an increased potential for retrievable information storage and bar-coding and combinatorial tagging (Benner and Sismour 2005). Naturally the four-letter genetic alphabet consists of just two pairs, A-T and G-C. In 2012, a genetic system comprising of three base pairs was introduced in (Malyshev et al. 2012). In addition to the natural base pairs, the third, unnatural or synthetic base pair 5SICS-MMO2 was proven to be functionally equivalent to a natural base pair. Moreover, when it is combined with the natural base pairs, 5SICS-MMO2 provides a fully functional six-letter genetic alphabet. Namely, six-letter genetic alphabets can be copied (Yang et al. 2007), polymerase chain reaction (PCR)-amplified and sequenced (Sismour et al. 2004; Yang et al. 2011), transcribed to six-letter RNA and back to six-letter DNA (Leal et al. 2015), and used to encode proteins with added amino acids (Bain et al. 1992). This biological importance and relevance of the above six-letter genetic alphabets motivates us to particularly study the $6\times 6$ Markov matrices describing the probabilities of changing base pairs in the six-letter genetic system in Sect. 6. When considering both DNA strands, each substitution is observed twice due to the complementarity between both strands, and hence the resulting transition matrix is centrosymmetric.

Moreover there are other synthetic analogs to natural DNA which justify studying centrosymmetric matrices for $n>6$. For instance, hachimoji DNA is a synthetic DNA that uses four synthetic nucleotides B,Z,P,S in addition to the four natural ones A,C,G,T. With the additional four synthetic ones, hachimoji DNA forms four types of base pairs, two of which are unnatural: P binds with Z and B binds with S. The complementarity between both strands of the DNA implies that the transition matrix is centrosymmetric. Moreover, the research group responsible for the hachimoji DNA system had also studied a synthetic DNA analog system that used twelve different nucleotides, including the four found in DNA (see Yang et al. 2006). Although the biological models which motivate the study of centrosymmetric matrices in this paper require *n* to be an even number due to the double-helix structure of DNA, in Sect. 5, we include the case of *n* being odd for completeness.

Apart from embeddability, that is existence of Markov generators, it is also natural to ask about uniqueness of a Markov generator which is called the rate identifiability problem. Identifiability is a property which a model must satisfy in order for precise statistical inference to be possible. A class of phylogenetic models is identifiable if any two models in the class produce different data distributions. In this article, we further develop the results on rate identifiability of the Kimura two parameter model (Casanellas et al. 2020a) to study rate identifiability for strand symmetric models. We also show that there are embeddable strand symmetric Markov matrices with non identifiable rates, namely the Markov generator is not unique. Moreover, we show that strand symmetric Markov matrices are not generically identifiable, that is, there exists a positive measure subset of strand symmetric Markov matrices containing embeddable matrices whose rates are not identifiable.

This paper is organised as follows. In Sect. 2, we introduce the basic definitions and results on embeddability. In Sect. 3, we give a characterisation for a $4 \times 4$ centrosymmetric Markov matrix *M* with four distinct real nonnegative eigenvalues to be embeddable providing necessary and sufficient conditions in Theorem 2, while we also discuss their rate identifiability property in Proposition 3. Moreover in Sect. 4, using the conditions of our main result Theorem 2, we compute the relative volume of all $4 \times 4$ centrosymmetric Markov matrices relative to the $4 \times 4$ centrosymmetric Markov matrices with positive eigenvalues and $\Delta >0$, as well as the relative volume of all $4 \times 4$ centrosymmetric Markov matrices relative to the $4 \times 4$ centrosymmetric Markov matrices with four distinct eigenvalues and $\Delta >0$. We also compare the results on relative volumes obtained using our method with the algorithm suggested in Casanellas et al. (2023) to showcase the advantages of our method. In Sect. 5, we study higher order centrosymmetric matrices and motivate their use in Sect. 6 by exploring the case of synthetic nucleotides where the phylogenetic models admit $6 \times 6$ centrosymmetric mutation matrices. Finally, Sect.  7 discusses implications and possibilities for future work.

## Preliminaries

In this section we will introduce the definitions and results that will be required throughout the paper. We will denote by $M_n({\mathbb {K}})$ the set of $n \times n$ square matrices with entries in the field ${\mathbb {K}}= {\mathbb {R}} \text { or } {\mathbb {C}}$. The subset of non-singular matrices in $M_n({\mathbb {K}})$ will be denoted by $GL_{n}({\mathbb {K}})$.

### Definition 1

A *Markov (or transition) matrix* is a non-negative real square matrix with rows summing to one. A *rate matrix* is a real square matrix with rows summing to zero and non-negative off-diagonal entries.

In this paper, we are focusing on a subset of Markov matrices called centrosymmetric Markov matrices.

### Definition 2

A real $n\times n$ matrix $A=(a_{i,j})$ is said to be *centrosymmetric (CS)* if

$$\begin{aligned} a_{i,j}=a_{n+1-i,n+1-j} \end{aligned}$$

for every $$1\le i,j\le n$$.

Definition 2 reveals that a CS matrix is nothing more than a square matrix which is symmetric about its center. This class of matrices has been previously studied, for instance, in (Aitken 2017, page 124) and Weaver (1985). Examples of CS matrices for $n=5$ and $n=6$, are the following two matrices respectively:

$$\begin{aligned} \begin{pmatrix} a_{11}&{}a_{12}&{}a_{13}&{}a_{14}&{} a_{15}\\ a_{21}&{}a_{22}&{}a_{23}&{}a_{24}&{}a_{25}\\ a_{31}&{}a_{32}&{}a_{33}&{}a_{32}&{}a_{31}\\ a_{25}&{}a_{24}&{}a_{23}&{}a_{22}&{}a_{21}\\ a_{15}&{}a_{14}&{}a_{13}&{}a_{12}&{}a_{11}\\ \end{pmatrix} \qquad \text { and } \qquad \begin{pmatrix} a_{11}&{}a_{12}&{}a_{13}&{}a_{14}&{} a_{15}&{}a_{16}\\ a_{21}&{}a_{22}&{}a_{23}&{}a_{24}&{}a_{25}&{}a_{26}\\ a_{31}&{}a_{32}&{}a_{33}&{}a_{34}&{}a_{35}&{}a_{36}\\ a_{36}&{}a_{35}&{}a_{34}&{}a_{33}&{}a_{32}&{}a_{31}\\ a_{26}&{}a_{25}&{}a_{24}&{}a_{23}&{}a_{22}&{}a_{21}\\ a_{16}&{}a_{15}&{}a_{14}&{}a_{13}&{}a_{12}&{}a_{11}\\ \end{pmatrix}. \end{aligned}$$

The class of CS matrices plays an important role in the study of Markov processes since they are indeed transition matrices for some processes in evolutionary biology. For instance, in Kimura (1957), centrosymmetric matrices are used to study the random assortment phenomena of subunits in chromosome division. Furthermore, in Schensted (1958), the same centrosymmetric matrices appear as the transition matrices in the model of subnuclear segregation in the macronucleus of ciliates. Finally, the work (Iosifescu 2014) examines a special case of the random genetic drift phenomenon, which consists of a population of individuals that are able to produce a single type of gamete. In this case, the transition matrices of the associated Markov chain are given by centrosymmetric matrices.

The embedding problem is directly related to the notions of matrix exponential and logarithm which we introduce for completeness below.

### Definition 3

We define the exponential $\exp (A)$ of a matrix *A*, using the Taylor power series of the function $f(x)=e^x$, as

$$\begin{aligned} \exp (A) = \sum _{k=0}^{\infty } \frac{A^k}{k!}, \end{aligned}$$

where $A^0= I_n$ and $I_n$ denotes the $n\times n$ identity matrix. If $A=P \;diag(\lambda _1,\dots ,\lambda _n)\;P^{-1}$ is an eigendecomposition of *A*, then $\exp (A) = P \;diag(e^{\lambda _1},\dots ,e^{\lambda _n})\;P^{-1}$. Given a matrix $A\in M_n({\mathbb {K}})$, a matrix $B\in M_n({\mathbb {K}})$ is said to be a *logarithm of* *A* if $\exp (B)=A$. If *v* is an eigenvector corresponding to the eigenvalue $\lambda$ of *A*, then *v* is an eigenvector corresponding to the eigenvalue $e^{\lambda }$ of $\exp (A)$.

A Markov matrix *M* is called *embeddable* if it can be written as the exponential of a rate matrix *Q*, namely $M= \exp (Q)$. Then any rate matrix *Q* satisfying the equation $M= \exp (Q)$ is called a *Markov generator of M*.

### Remark 1

Embeddable Markov matrices occur when we assume a continuous time Markov chain, in which case the Markov matrices have the form

$$\begin{aligned} M= \exp (t Q), \end{aligned}$$

where $t \ge 0$ represents time and *Q* is a rate matrix. However, in the rest of the paper, we assume that *t* is incorporated in the rate matrix *Q*.

The existence of multiple logarithms is a direct consequence of the distinct branches of the logarithmic function in the complex field.

### Definition 4

Given $z\in {\mathbb {C}}\setminus {\mathbb {R}}_{\le 0}$ and $k\in {\mathbb {Z}}$, the *k*-th branch of the logarithm of *z* is $\log _k(z):= \log |z| + (Arg(z)+2\pi k)i$, where $\log$ is the logarithmic function on the real field and $Arg(z)\in (-\pi ,\pi )$ denotes the principal argument of *z*. The logarithmic function arising from the branch $\log _0(z)$ is called the principal logarithm of *z* and is denoted as $\log (z)$.

It is known that if *A* is a matrix with no negative eigenvalues, then there is a unique logarithm of *A* all of whose eigenvalues are given by the principal logarithm of the eigenvalues of *A* (Higham 2008, Theorem 1.31). We refer to this unique logarithm as the *principal logarithm of* *A*, denoted by *Log*(*A*).

By definition, the Markov generators of a Markov matrix *M* are those logarithms of *M* that are rate matrices. In particular they are real logarithms of *M*. The following result enumerates all the real logarithms with rows summing to zero of any given Markov matrix with positive determinant and distinct eigenvalues. Therefore, all Markov generators of such a matrix are necessarily of this form.

### Proposition 1

(Casanellas et al. 2023, Proposition 4.3). Let $M=P\; diag \big ( 1,\lambda _1,\dots ,\lambda _t, \mu _1,\overline{\mu _1},\dots ,\mu _s,\overline{\mu _s} \big ) \; P^{-1}$ be an $n\times n$ Markov matrix with $P\in GL_n({\mathbb {C}})$ and distinct eigenvalues $\lambda _i\in {\mathbb {R}}_{>0}$ for $i=1,\dots ,t$ and $\mu _j \in \{z\in {\mathbb {C}}: Im(z) > 0\}$ for $j=1,\dots ,s$, all of them pairwise distinct. Then, a matrix *Q* is a real logarithm of *M* with rows summing to zero if and only if $Q=P\; diag \Big ( 0, \log (\lambda _1),\dots ,\log (\lambda _t), \log _{k_1}(\mu _1),\overline{\log _{k_1}(\mu _1)},\dots ,\log _{k_s}(\mu _s),\overline{\log _{k_s}(\mu _s)} \Big ) \; P^{-1}$ for some $k_1,\dots ,k_j\in {\mathbb {Z}}$.

### Remark 2

In particular, the principal logarithm of *M* can be computed as

$$\begin{aligned} Log(M) = P\; diag \Big ( 0, \log (\lambda _1),\dots ,\log (\lambda _t), \log (\mu _1),\log (\overline{\mu _1}),\dots ,\log (\mu _s),\log (\overline{\mu _s}) \Big ) \; P^{-1}. \end{aligned}$$

In this paper, we focus on the embedding problem for the class of centrosymmetric matrices. In Sect. 3, we will first study the embeddability of $4\times 4$ centrosymmetric Markov matrices, which include the K3P, K2P and JC Markov matrices. In Sect. 5 and Sect. 6, we will further study the embeddability of higher order centrosymmetric Markov matrices.

## Embeddability of $4\times 4$ centrosymmetric matrices

In this section, we begin our study by analyzing the embeddability of $4\times 4$ centrosymmetric matrices also known as strand symmetric matrices. We will provide necessary and sufficient conditions for $4\times 4$ centrosymmetric matrices to be embeddable. Moreover, we will discuss their rate identifiability problem as well.

The transition matrices of $4\times 4$ centrosymmetric matrices are assumed to have the form

$$\begin{aligned}M= \begin{pmatrix} m_{11}&{}m_{12}&{}m_{13}&{}m_{14}\\ m_{21}&{}m_{22}&{}m_{23}&{}m_{24}\\ m_{24}&{}m_{23}&{}m_{22}&{}m_{21}\\ m_{14}&{}m_{13}&{}m_{12}&{}m_{11}\\ \end{pmatrix}, \end{aligned}$$

where

$$\begin{aligned} m_{11}+m_{12}+m_{13}+m_{14}=1=m_{21}+m_{22}+m_{23}+m_{24} \text{ and } m_{ij}\ge 0. \end{aligned}$$

Recall that the K3P matrices are assumed to have the form

$$\begin{aligned} M= \begin{pmatrix} m_{11}&{}m_{12}&{}m_{13}&{}m_{14}\\ m_{12}&{}m_{11}&{}m_{14}&{}m_{13}\\ m_{13}&{}m_{14}&{}m_{11}&{}m_{12}\\ m_{14}&{}m_{13}&{}m_{12}&{}m_{11}\\ \end{pmatrix}. \end{aligned}$$

In the case of the K2P matrices, we additionally have $m_{12} = m_{13}$, while in the case of JC matrices, $m_{12} = m_{13} = m_{14}$. It can be easily seen that K3P, K2P, and JC Markov (rate) matrices are centrosymmetric.

Let us define the following matrix

3.1 $$\begin{aligned} S=\begin{pmatrix} 1&{}0&{}0&{}1\\ 0&{}1&{}1&{}0\\ 0&{}1&{}-1&{}0\\ 1&{}0&{}0&{}-1 \end{pmatrix}; \end{aligned}$$

compare (Casanellas and Kedzierska 2013, Section 6). For a $4\times 4$ CS Markov matrix *M*, we define $F(M):=S^{-1}M S$. By direct computation, it can be checked that *F*(*M*) is a block diagonal matrix

3.2 $$\begin{aligned} F(M)= \begin{pmatrix} \lambda &{}1-\lambda &{}0&{}0\\ 1-\mu &{}\mu &{}0&{}0\\ 0&{}0&{}\alpha &{}\alpha '\\ 0&{}0&{}\beta '&{}\beta \\ \end{pmatrix}, \end{aligned}$$

where

3.3 $$\begin{aligned} \begin{aligned} \lambda =m_{11}+m_{14},\ {}&\qquad&\mu =m_{22}+m_{23},\\ \alpha =m_{22}-m_{23},\ {}&\qquad&\alpha '=m_{21}-m_{24},\\ \beta =m_{11}-m_{14},\ {}&\qquad&\beta '=m_{12}-m_{13}. \end{aligned} \end{aligned}$$

Define two matrices, $$M_1:=\begin{pmatrix}\lambda &{}1-\lambda \\ 1-\mu &{}\mu \\ \end{pmatrix}$$ and $$M_2:=\begin{pmatrix}\alpha &{}\alpha '\\ \beta '&{}\beta \\ \end{pmatrix}$$, which are the upper and lower block matrices in (3.2), respectively.

Similarly, the rate matrices in strand symmetric models are assumed to have the $4 \times 4$ centrosymmetric form

$$\begin{aligned}Q= \begin{pmatrix} q_{11}&{}q_{12}&{}q_{13}&{}q_{14}\\ q_{21}&{}q_{22}&{}q_{23}&{}q_{24}\\ q_{24}&{}q_{23}&{}q_{22}&{}q_{21}\\ q_{14}&{}q_{13}&{}q_{12}&{}q_{11}\\ \end{pmatrix}, \end{aligned}$$

where

$$\begin{aligned} q_{11}+q_{12}+q_{13}+q_{14}=0=q_{21}+q_{22}+q_{23}+q_{24} \text{ and } q_{ij}\ge 0 \text{ for } i\ne j. \end{aligned}$$

So, for a $4\times 4$ CS rate matrix *Q*, we can also define $F(Q):=S^{-1}Q S$. By direct computation, it can be checked that

3.4 $$\begin{aligned} F(Q)= \begin{pmatrix} -\rho &{}\rho &{}0&{}0\\ \sigma &{}-\sigma &{}0&{}0\\ 0&{}0&{}\delta &{}\delta '\\ 0&{}0&{}\gamma '&{}\gamma \\ \end{pmatrix}, \end{aligned}$$

where

$$\begin{aligned} \rho =q_{12}+q_{13},&\sigma =q_{21}+q_{24},\\ \delta =q_{22}-q_{23},&\delta '=q_{21}-q_{24},\\ \gamma =q_{11}-q_{14},&\gamma '=q_{12}-q_{13}. \end{aligned}$$

Define two matrices, $$Q_1:=\begin{pmatrix}-\rho &{}\rho \\ \sigma &{}-\sigma \\ \end{pmatrix}$$ and $$Q_2:=\begin{pmatrix}\delta &{}\delta '\\ \gamma '&{}\gamma \\ \end{pmatrix}$$, which are the upper and lower block matrices in (3.4), respectively.

The following results provide necessary conditions for a $4\times 4$ CS Markov matrix to be embeddable.

### Lemma 1

Let $M=(m_{ij})$ be a $4\times 4$ CS Markov matrix and $M=\exp (Q)$ for some CS rate matrix *Q*. Then

1. $$m_{11}+m_{14}+m_{22}+m_{23}>1$$ and
2. $$(m_{22}-m_{23})(m_{11}-m_{14})>(m_{24}-m_{21})(m_{13}-m_{12}).$$

### Proof

We have that

$$\begin{aligned} F(M)=S^{-1}MS=S^{-1} \exp {(Q)} S=\exp (S^{-1}QS)=\exp {(F(Q))}. \end{aligned}$$

Then

$$\begin{aligned} \begin{pmatrix} M_1&{}0\\ 0&{}M_2\\ \end{pmatrix} =\exp (F(Q))=\begin{pmatrix} \exp (Q_1)&{}0\\ 0&{} \exp (Q_2)\\ \end{pmatrix}. \end{aligned}$$

Thus, $M_1$ is an embeddable $2\times 2$ Markov matrix. Using the embeddability criteria of $2\times 2$ Markov matrices in Kingman (1962), we have that $1<tr(M_1)=\lambda +\mu$, which is the desired inequality. Additionally, since $M_2=\exp (Q_2)$, $det(M_2)>0$ as desired. $\square$

### Lemma 2

Let $M=(m_{ij})$ be a $4\times 4$ CS Markov matrix and $M=\exp (Q)$ for some CS rate matrix $Q=(q_{ij})$. If $\lambda +\mu \ne 2$, then

$$\begin{aligned} q_{12}+q_{13}=\frac{-\lambda +1}{\lambda +\mu -2}\ln (\lambda +\mu -1) \end{aligned}$$

and

$$\begin{aligned} q_{21}+q_{24}=\frac{-\mu +1}{\lambda +\mu -2}\ln (\lambda +\mu -1). \end{aligned}$$

### Proof

By direct computations and the proof of Lemma 1,

$$\begin{aligned} M_1=\exp (Q_1)=\frac{1}{\rho +\sigma }\begin{pmatrix} e^{-\rho -\sigma }\rho +\sigma &{}-e^{-\rho -\sigma }\rho +\rho \\ -e^{-\rho -\sigma }\sigma +\sigma &{}e^{-\rho -\sigma }\sigma +\rho \\ \end{pmatrix}. \end{aligned}$$

We then have the following system of equations:

3.5 $$\begin{aligned} \lambda =\frac{e^{-\rho -\sigma }\rho +\sigma }{\rho +\sigma } \text{ and } \mu =\frac{e^{-\rho -\sigma }\sigma +\rho }{\rho +\sigma }. \end{aligned}$$

Summing the two equations, we get

$$\begin{aligned} \lambda +\mu =e^{-\rho -\sigma }+1. \end{aligned}$$

Note that by Lemma 1, $\lambda +\mu >1.$ Therefore,

3.6 $$\begin{aligned} \rho +\sigma =-\ln (\lambda +\mu -1). \end{aligned}$$

Using Equation (3.5) and (3.6), we obtain

$$\begin{aligned} \rho =\frac{-\lambda +1}{\lambda +\mu -2}\ln (\lambda +\mu -1)\qquad \text{ and }\qquad \sigma =\frac{-\mu +1}{\lambda +\mu -2}\ln (\lambda +\mu -1). \end{aligned}$$

The proof is now complete. $\square$

### Proposition 2

Given two matrices $A=(a_{ij}),B=(b_{ij}) \in M_2({\mathbb {R}})$, consider the block-diagonal matrix $C= diag(A,B)$. Then the following statements hold:

i) $F^{-1}(C):= S C S^{-1}$ is a CS matrix.
ii) $F^{-1}(C)$ is a Markov matrix if and only if *A* is a Markov matrix and
  $$\begin{aligned} |b_{2 2}|\le a_{1 1}, \qquad |b_{2 1}|\le a_{1 2}, \qquad |b_{1 2}|\le a_{ 2 1}, \qquad |b_{1 1}|\le a_{2 2}. \end{aligned}$$
iii) $F^{-1}(C)$ is a rate matrix if and only if *A* is a rate matrix and
  $$\begin{aligned} b_{2 2}\le a_{1 1}(\le 0), \quad |b_{2 1}|\le a_{1 2} (=-a_{1 1}), \quad |b_{1 2}|\le a_{2 1}(=-a_{2 2}), \quad b_{1 1}\le a_{2 2}(\le 0). \end{aligned}$$

### Proof

To prove i), by direct computation we obtain that

$$\begin{aligned} F^{-1}(C)= S C S^{-1} = \frac{1}{2}\begin{pmatrix} a_{1 1}+b_{2 2} &{} a_{1 2}+b_{2 1} &{} a_{1 2} - b_{2 1} &{} a_{1 1} - b_{2 2}\\ a_{2 1}+b_{1 2} &{} a_{2 2} + b_{1 1} &{} a_{2 2} - b_{1 1} &{} a_{2 1} -b_{1 2}\\ a_{2 1} - b_{1 2} &{} a_{2 2} - b_{1 1} &{} a_{2 2} + b_{1 1} &{} a_{2 1}+b_{1 2} \\ a_{1 1}-b_{2 2} &{} a_{1 2} -b_{2 1} &{} a_{1 2} +b_{2 1} &{} a_{1 1} + b_{2 2}\\ \end{pmatrix}. \end{aligned}$$

Then ii) follows from the above expression of $F^{-1}(Q)$ and the fact that rows of Markov matrices add to 1 and the entries are non-negative, while iii) similarly follows from the fact that the rows of rate matrices add to zero and the off-diagonal entries are non-negative.$\square$

For any $4\times 4$ CS Markov matrix $M=(m_{ij})$, let us recall that by (3.2), *M* is block-diagonalizable via the matrix *S*. In the rest of this section, we will study both the upper and the lower block matrices of *F*(*M*) more closely. Studying the upper and lower blocks allows us to establish the main result of the embeddability criteria for $4\times 4$ CS Markov matrices. This block-diagonalization reduces our analysis to studying the logarithms of both the upper and the lower block matrices which have size $2\times 2$. This result will be presented in Theorem 2.

### Upper block

As we have seen in (3.2), the upper block of *F*(*M*) is given by the $2\times 2$ matrix $$M_1=\begin{pmatrix} \lambda &{} 1-\lambda \\ 1-\mu &{}\mu \\ \end{pmatrix}$$, which is a Markov matrix. If $$P_1=\begin{pmatrix} 1&{}1-\lambda \\ 1&{}\mu -1\\ \end{pmatrix}$$, then

$$\begin{aligned} P_1^{-1}M_1P_1=\begin{pmatrix} 1&{}0\\ 0&{}\lambda +\mu -1\\ \end{pmatrix}. \end{aligned}$$

Hence, by Proposition 1, any logarithm of $M_1$ can be written as

$$\begin{aligned} L^{M_1}_{k_1,k_2}:=P_1\begin{pmatrix} 2k_1\pi i&{}0\\ 0&{}\log (\lambda +\mu -1)+2k_2\pi i\\ \end{pmatrix}P_1^{-1}, \end{aligned}$$

for some integers $k_1$ and $k_2$. Let $p=\log (\lambda +\mu -1)$, $q=1-\lambda$, and $r=1-\mu$. Then

3.7 $$\begin{aligned} L^{M_1}_{k_1,k_2}=\frac{1}{2-\lambda -\mu }\begin{pmatrix} qp+2\pi (rk_1+qk_2)i&{}-qp+2\pi q(k_1-k_2)i\\ -rp+2\pi r(k_1-k_2)i&{}rp+2\pi (qk_1+rk_2)i\\ \end{pmatrix}. \end{aligned}$$

#### Lemma 3

If $\lambda +\mu \ne 2$, then $L^{M_1}_{k_1,k_2}$ is a real matrix if and only if $k_1=k_2=0$ and $\lambda +\mu >1$. In this case, the only real logarithm of $M_1$ is the principal logarithm

$$\begin{aligned} \frac{1}{2-\lambda -\mu }\begin{pmatrix} qp&{}-qp\\ -rp&{}rp\\ \end{pmatrix}. \end{aligned}$$

#### Proof

For fixed $k_1$ and $k_2$, the eigenvalues of $L^{M_1}_{k_1,k_2}$ are $\lambda _1=2k_1\pi i$ and $\lambda _2=p+2k_2\pi i.$ Then $L^{M_1}_{k_1,k_2}$ is a real matrix if and only if $\lambda _1,\lambda _2\in {\mathbb {R}}$ or $\lambda _2=\overline{\lambda _1}$. Since $\lambda +\mu \ne 2$, $\lambda _2\ne \overline{\lambda _1}$. Thus, $L^{M_1}_{k_1,k_2}$ is a real matrix if and only if $\lambda _1,\lambda _2\in {\mathbb {R}}$. Finally, $\lambda _1\in {\mathbb {R}}$ if and only if $k_1=0$ and $\lambda _2\in {\mathbb {R}}$ if and only if $k_2=0$ and $\lambda +\mu >1$. $\square$

### Lower block

The lower block of *F*(*M*) is given by the matrix $$M_2=\begin{pmatrix} \alpha &{}\alpha '\\ \beta '&{}\beta \\ \end{pmatrix}$$. Unlike $M_1$, the matrix $M_2$ is generally not a Markov matrix. The discriminant of the characteristic polynomial of $M_2$ is given by

3.8 $$\begin{aligned} \Delta :=(\alpha -\beta )^2+4\alpha '\beta ' \end{aligned}$$

with $\alpha ,\beta ,\alpha ',\beta '$ defined as in (3.3). If $\Delta >0$, then $M_2$ has two distinct real eigenvalues and if $\Delta <0$, then $M_2$ has a pair of conjugated complex eigenvalues. Moreover, if $\Delta =0$, then $M_2$ has either $2\times 2$ Jordan block or a repeated real eigenvalue. We will assume that $\Delta \ne 0$ so that $M_2$ diagonalizes into two distinct eigenvalues.

Let $$P_2=\begin{pmatrix} \frac{\sqrt{\Delta }+(\alpha -\beta )}{2}&{}\frac{\sqrt{\Delta }-(\alpha -\beta )}{2}\\ \beta '&{}-\beta '\\ \end{pmatrix}$$. Then

$$\begin{aligned} P_2^{-1}M_2P_2=\begin{pmatrix} \frac{(\alpha +\beta )+\sqrt{\Delta }}{2}&{}0\\ 0&{}\frac{(\alpha +\beta )-\sqrt{\Delta }}{2}\\ \end{pmatrix}. \end{aligned}$$

Let us now define

$$\begin{aligned} l_3:=\log (\frac{(\alpha +\beta )+\sqrt{\Delta }}{2})+2k_3\pi i\quad \text{ and }\quad l_4:=\log (\frac{(\alpha +\beta )-\sqrt{\Delta }}{2})+2k_4\pi i, \end{aligned}$$

where $k_3$ and $k_4$ are integers. Therefore, any logarithm of $M_2$ can be written as

3.9 $$\begin{aligned} L^{M_2}_{k_3,k_4}:=\begin{pmatrix} \varepsilon &{}\phi \\ \gamma &{}\eta \\ \end{pmatrix} \end{aligned}$$

where

$$\begin{aligned} \varepsilon&:=\frac{1}{2}((l_3+l_4)+(\alpha -\beta )\frac{(l_3-l_4)}{\sqrt{\Delta }}),\\ \phi&:=\alpha '\frac{(l_3-l_4)}{\sqrt{\Delta }},\\ \gamma&:=\beta '\frac{(l_3-l_4)}{\sqrt{\Delta }} \text{ and } \\ \eta&:=\frac{1}{2}((l_3+l_4)-(\alpha -\beta )\frac{(l_3-l_4)}{\sqrt{\Delta }}). \end{aligned}$$

#### Lemma 4

1. If $\Delta >0$, then $L^{M_2}_{k_3,k_4}$ is a real matrix if and only if $\alpha +\beta >\sqrt{\Delta }$ and $k_3=k_4=0.$
2. If $\Delta <0$, then $L^{M_2}_{k_3,k_4}$ is a real matrix if and only if $k_4=-k_3$.

#### Proof

1. If $\Delta >0$, then $Im(l_3)=2k_3\pi$ and $Im(l_4)=2k_4\pi$. Moreover, $Re(l_3)\ne Re(l_4).$ Since $l_3$ and $l_4$ are the eigenvalues of $L^{M_2}_{k_3,k_4}$, this implies that $l_3\ne {\overline{l}}_4$. In particular, $L^{M_2}_{k_3,k_4}$ is a real matrix if and only if both $l_3$ and $l_4$ are real.
2. Let us assume $\Delta <0$ and take $z=\frac{(\alpha +\beta )+\sqrt{\Delta }}{2}$. Fixing $k_3,k_4\in {\mathbb {Z}}$, the eigenvalues of $L^{M_2}_{k_3,k_4}$ are $l_3=\log (z)+2k_3\pi i$ and $l_4=\log ({\overline{z}})+2k_4\pi i=\overline{log(z)}+2k_4\pi i$, which are both complex numbers. Thus, $L^{M_2}_{k_3,k_4}$ is real if and only if $l_3={\overline{l}}_4$. Hence, $k_4=-k_3.$ Conversely, $k_4=-k_3$ implies that $l_3+l_4=2 Re(l_3)\in {\mathbb {R}}$ and $\frac{l_3-l_4}{\sqrt{\Delta }}=\frac{2 Im(l_3)i}{\sqrt{\Delta }}\in {\mathbb {R}}$. Thus, all entries of $L^{M_2}_{k_3,k_4}$ are real.

$$\square $$

### Logarithms of $4\times 4$ CS Markov matrices

Let *M* be a $4\times 4$ CS Markov matrix. Using the values defined in (3.3) and (3.8), we can now label up its four eigenvalues as following,

3.10 $$\begin{aligned} 1,\quad \lambda _1:=\lambda +\mu -1,\quad \lambda _2:=\frac{(\alpha +\beta )+\sqrt{\Delta }}{2}\quad \text{ and } \quad \lambda _3=\frac{(\alpha +\beta )-\sqrt{\Delta }}{2}. \end{aligned}$$

We note that the subset of $4\times 4$ CS Markov matrix with repeated eigenvalues (diagonalizing matrix with repeated eigenvalues or a Jordan block of size greater than 1) have zero measure. Therefore generic $4\times 4$ Markov matrices have no repeated eigenvalues, and hence we are going to assume the eigenvalues to be distinct. In particular, we are assuming that *M* diagonalizes. Furthermore, since we want *M* to have real logarithms and have no repeated eigenvalues, we need the real eigenvalues to be positive.

The following theorem characterizes the embeddability of a $4\times 4$ CS Markov matrix with positive and distinct eigenvalues. Furthermore, the theorem guarantees that a $4\times 4$ CS Markov matrix is embeddable if and only if it admits a CS Markov generator. In particular, the characterization of the embeddability of a CS matrix is equivalent when restricting to rate matrices satisfying the symmetries imposed by the model (model embeddability) than when restricting to all possible rate matrices (embedding problem).

#### Theorem 1

Let *M* be a diagonalizable $4\times 4$ CS Markov matrix with positive and distinct eigenvalues $\lambda _1, \lambda _2, \lambda _3$ defined as in (3.10). Let us define

$$\begin{aligned} x=\log (\lambda _1),\qquad y_k=\log (\lambda _2)+2k\pi i,\qquad z_k=\log (\lambda _3)-2k\pi i, \end{aligned}$$

where $k=0$ if $\Delta >0$ and $k\in {\mathbb {Z}}$ if $\Delta <0.$ Then any real logarithm of *M* is given by

$$\begin{aligned} S\begin{pmatrix} \alpha _1&{}-\alpha _1&{}0&{}0\\ -\beta _1&{}\beta _1&{}0&{}0\\ 0&{}0&{}\delta (k)&{}\varepsilon (k)\\ 0&{}0&{}\phi (k)&{}\gamma (k)\\ \end{pmatrix}S^{-1}, \end{aligned}$$

where

$$\begin{aligned} \alpha _1&= \frac{1-\lambda }{2-\lambda -\mu }x,\qquad&\beta _1&=\frac{1-\mu }{2-\lambda -\mu }x,\\ \delta (k)&=\frac{1}{2}((y_k+z_k)+(\alpha -\beta )\frac{(y_k-z_k)}{\sqrt{\Delta }}),\qquad&\varepsilon (k)&=\alpha '\frac{(y_k-z_k)}{\sqrt{\Delta }},\\ \phi (k)&=\beta '\frac{(y_k-z_k)}{\sqrt{\Delta }},\qquad&\gamma (k)&=\frac{1}{2}((y_k+z_k)-(\alpha -\beta )\frac{(y_k-z_k)}{\sqrt{\Delta }}). \end{aligned}$$

with $\lambda ,\ \mu ,\ \alpha ,\ \beta ,\ \alpha ' \text { and } \beta '$ defined as in (3.3) and $\Delta$ as in (3.8).

In particular, any real logarithm of *M* is also a $4\times 4$ CS matrix whose entries $q_{11},\dots ,q_{24}$ are given by:

$$\begin{aligned}&q_{11}=\frac{\alpha _1+\gamma (k)}{2},\quad&q_{12}=\frac{-\alpha _1+\phi (k)}{2},\quad&q_{13}=\frac{-\alpha _1-\phi (k)}{2},\quad&q_{14}=\frac{\alpha _1-\gamma (k)}{2},\\&q_{21}=\frac{-\beta _1+\varepsilon (k)}{2},\quad&q_{22}=\frac{\beta _1+\delta (k)}{2},\quad&q_{23}=\frac{\beta _1-\delta (k)}{2},\quad&q_{24}=\frac{-\beta _1-\varepsilon (k)}{2}. \end{aligned}$$

#### Proof

Let us note that

$$\begin{aligned} M=S\cdot \text{ diag }(P_1,P_2)\cdot \text{ diag }(1, \lambda _1, \lambda _2, \lambda _3)\cdot \text{ diag }(P_1^{-1},P_2^{-1})\cdot S^{-1}. \end{aligned}$$

Since we assume that the eigenvalues of *M* are distinct, according to Proposition 1, any logarithm of *M* can be written as

$$\begin{aligned} Q&=S\cdot \text{ diag }(P_1,P_2)\cdot \text{ diag }(\log _{k_1}(1), \log _{k_2}(\lambda _1),\log _{k_3}(\lambda _2),\log _{k_4}(\lambda _3))\cdot \text{ diag }(P_1^{-1},P_2^{-1})\cdot S^{-1}\\&=S\cdot \text{ diag }(L^{M_1}_{k_1,k_2},L^{M_2}_{k_3,k_4})\cdot S^{-1}, \end{aligned}$$

The last equation and the fact that *S* and $S^{-1}$ are real matrices imply that *Q* will be real if and only if both $L^{M_1}_{k_1,k_2}$ and $L^{M_2}_{k_3,k_4}$ are real. Here $L^{M_1}_{k_1,k_2}$ is the upper block given in (3.7) and $L^{M_2}_{k_3,k_4}$ is the lower block defined in (3.9). By Lemma 3, $L^{M_1}_{k_1,k_2}$ being a real logarithm implies that $k_1=k_2=0$ and $\lambda +\mu >1$. Then $L^{M_2}_{k_3,k_4}$ being a real matrix, according to Lemma 4, implies that $k_3=k_4=0$ if $\Delta >0$, while $k_4=-k_3$ if $\Delta <0.$ Therefore, the upper block is $L^{M_1}_{0,0}$ and the lower block will be $L^{M_2}_{k,-k},\text { for } k=k_3$ completing the proof. $\square$

Now we are interested in knowing when the real logarithm of a $4\times 4$ CS Markov matrix is a rate matrix. Using the same notation as in Theorem 1 we get the following result.

#### Theorem 2

A diagonalizable $4\times 4$ CS Markov matrix *M* with distinct eigenvalues is embeddable if and only if the following conditions hold for $k=0$ if $\Delta >0$ or for some $k\in {\mathbb {Z}}$ if $\Delta <0$:

$$\begin{aligned} \lambda _1>0,\quad (\alpha +\beta )^2>\Delta ,\quad |\phi (k)|\le -\alpha _1,\quad |\varepsilon (k)|\le -\beta _1,\quad \gamma (k)\le \alpha _1,\quad \delta (k)\le \beta _1. \end{aligned}$$

#### Proof

The logarithm of a $4\times 4$ CS Markov matrix will depend on whether $\Delta >0$ or $\Delta <0$. In particular, it will depend on whether the eigenvalues $\lambda _2$ and $\lambda _3$ are real and positive or whether they are conjugated complex numbers.

1. If $\Delta >0$, then both $\lambda _2$ and $\lambda _3$ are real and $\lambda _2>\lambda _3$. Hence, $z<y<0.$ Moreover, Lemma 4 implies that $\lambda _3>0$ and hence $\lambda _2\lambda _3>0$.
2. If $\Delta <0$, then $\lambda _2,\lambda _3\in {\mathbb {C}}\setminus {\mathbb {R}}$ and $\lambda _2=\overline{\lambda _3}$. Hence, $y+z>0$ and $y-z=4\pi ki.$ Moreover, $\lambda _2\lambda _3=|\lambda _3|^2>0$ since $\lambda _3\ne 0$.

Thus, in both cases, $\alpha _1,\beta _1,\delta (k),\varepsilon (k), \phi (k),\gamma (k)\in {\mathbb {R}}.$ Moreover, $\alpha _1$ and $\beta _1$ are both non-positive. In particular, Theorem 1 together with Proposition 2 imply that a real logarithm of *M* is a rate matrix if and only if

$$\begin{aligned} |\phi (k)|\le -\alpha _1,\qquad |\varepsilon (k)|\le -\beta _1,\qquad \gamma (k)\le \alpha _1,\qquad \delta (k)\le \beta _1. \end{aligned}$$

Furthermore, the conditions $\lambda _1>0$ comes from Lemma 3. The proof is now complete. $\square$

#### Remark 3

According to Theorem 2 the embeddability of a $4\times 4$ CS Markov matrix *M* with distinct positive eigenvalues can be decided by checking six inequalities depending on the entries of *M*. However, if *M* has non-real eigenvalues then one has to check infinitely many groups of inequalities, one for each value of $k\in {\mathbb {Z}}$. It is enough that one of those systems is consistent to guarantee that *M* is embeddable. Theorem 5.5 in Casanellas et al. (2023) provides boundaries for the values of *k* for which the corresponding inequalities may hold.

Let us take a look at the class of K3P matrices which is a special case of strand symmetric matrices. Indeed, for a K3P matrix $M=(m_{ij})$, we have that

$$\begin{aligned} m_{11}=m_{22},\qquad m_{12}=m_{21},\qquad m_{13}=m_{24}\qquad \text{ and }\qquad m_{14}=m_{23}. \end{aligned}$$

Suppose that a K3P-Markov matrix $M=(m_{ij})$ is K3P-embeddable, i.e. $M=\exp (Q)$ for some K3P-rate matrix *Q*. Recall that the eigenvalues of *M* are

$$\begin{aligned}{} & {} 1,\quad p:=m_{11}+m_{12}-m_{13}-m_{14},\\{} & {} \quad \quad q:=m_{11}-m_{12}+m_{13}-m_{14}\quad \text{ and } \quad r:=m_{11}-m_{12}-m_{13}+m_{14}. \end{aligned}$$

In this case, we have that

$$\begin{aligned}&\lambda =\mu =m_{11}+m_{14},\quad \alpha =\beta =m_{11}-m_{14},\quad \alpha '=\beta '=m_{13}-m_{12},\quad \\&\lambda _1=r\quad \text{ and } \quad \Delta =4(m_{13}-m_{12})^2. \end{aligned}$$

In particular, we see that $\Delta > 0$ unless $m_{12}=m_{13}$. Moreover,

$$\begin{aligned}&x=\log r,\quad y=\log q,\quad z=\log p,\quad \alpha _1=\beta _1=\frac{1}{2}\log r,\\&\quad \delta (0)=\gamma (0)=\frac{1}{2}\log pq,\quad |\varepsilon (0)|=|\phi (0)|=\frac{1}{2}\log \frac{q}{p}. \end{aligned}$$

The inequalities in Theorem 2 can be spelled out as follows:

$$\begin{aligned} r>0,\quad pq>0,\quad |\log \frac{q}{p}|\le -\log r\quad \text{ and }\quad \log pq\le \log r. \end{aligned}$$

These inequalities are equivalent to the K3P-embeddability criteria presented in (Roca-Lacostena and Fernández-Sánchez 2018b, Theorem 3.1) and (Ardiyansyah et al. 2021, Theorem 1). Moreover, they are also equivalent to the restriction to centrosymmetric-matrices of the embeddability criteria for $4\times 4$ Markov matrices with different eigenvalues given in (Casanellas et al. 2023, Theorem 1.1)

In the last part of this section, we discuss the rate identifiability problem for $4\times 4$ centrosymmetric matrices. If a centrosymmetric Markov matrix arises from a continuous-time model, then we want to determine its corresponding substitution rates. In other words, given an embeddable $4\times 4$ CS matrix, we want to know if we can uniquely identify its Markov generator.

It is worth noting that Markov matrices with repeated real eigenvalues may admit more than one Markov generator (e.g. examples 4.2 and 4.3 in (Casanellas et al. 2020a) show embeddable K2P matrices with more than one Markov generator). Nonetheless, this is not possible if the Markov matrix has distinct eigenvalues, because in this case its only possible real logarithm would be the principal logarithm (Culver 1966). As one considers less restrictions in a model, the measure of the set of matrices with repeated real eigenvalues decreases, eventually becoming a measure zero set. For example, this is the case within the K3P model, where both its submodels (the K2P model and the JC model) consist of matrices with repeated eigenvalues and have positive measure subsets of embeddable matrices with non-identifiable rates. However, when considering the whole set of K3P Markov matrices, the subset of embeddable matrices with more than one Markov generator has measure zero (see Chapter 4 in (Roca-Lacostena 2021)). Nevertheless, this behaviour only holds if the Markov matrices within the model have real eigenvalues.

#### Proposition 3

There is a positive measure subset of $4\times 4$ CS Markov matrices that are embeddable and whose rates are not identifiable. Moreover, all the Markov generators of the matrices in this set are also CS matrices.

#### Proof

Given

$$\begin{aligned} P= \begin{pmatrix}\begin{array}{cccc} 1 &{}\qquad -5 &{}\qquad 1-i &{}\qquad 1+i\\ 1 &{}\qquad 2 &{}\qquad -i &{}\qquad i\\ 1 &{}\qquad 2 &{}\qquad i &{}\qquad -i\\ 1 &{}\qquad -5 &{}\qquad -1+i &{}\qquad -1-i\\ \end{array} \end{pmatrix}, \end{aligned}$$

let us consider the following matrices

$$\begin{aligned}{} & {} M= P \;diag(1, e^{-7\pi }, e^{-4\pi }i, - e^{-4\pi }i)\; P^{-1}, Q= P\; diag(0, -7\pi , -4\pi -\frac{3\pi }{2}i, -4\pi +\frac{3\pi }{2}i)\; P^{-1}. \end{aligned}$$

A straightforward computation shows that *M* is a CS Markov matrix and *Q* is a CS rate matrix. Moreover they both have non-zero entries. By applying the exponential series to *Q*, we get that $\exp (Q)=M$. This means that *M* is embeddable and *Q* is a Markov generator of *M*.

Since *Q* is a rate matrix, so is *Qt* for any $t\in {\textbf{R}}_{\ge 0}$. Therefore, $\exp (Qt)$ is an embeddable Markov matrix, because the exponential of any rate matrix is necessarily a Markov matrix. See (Pachter and Sturmfels 2005, Theorem 4.19) for more details. Moreover, we have that

$$\begin{aligned} S^{-1}P = \begin{pmatrix} 1 &{} -5 &{} 0 &{}0 \\ 1 &{} 2 &{}0 &{} 0\\ 0&{}0 &{} -i &{} i\\ 0 &{} 0&{} 1-i &{} 1+i\\ \end{pmatrix}, \end{aligned}$$

so $S^{-1} \exp (Qt)S$ is a 2-block diagonal matrix. Hence, by Proposition 2 we have that $\exp (Qt)$ is an embeddable strand symmetric Markov matrix for all $t\in {\mathbb {R}}_{>0}$.

Now, let us define $V=P\;diag(0,0,2\pi i, -2\pi i)\;P^{-1}$. Note that *Q* and *V* diagonalize simultaneously via *P* and hence they commute. Therefore,

$$\begin{aligned} \exp (Q+V)= \exp (Q)\exp (V)=M I_4= M \end{aligned}$$

by the Baker-Campbell-Haussdorff formula. Moreover,

$$\begin{aligned} \exp (Qt+kV)= \exp (Qt)\exp (kV)=\exp (Qt)I_4= \exp (Qt) \end{aligned}$$

for all $k \in {\mathbb {Z}}$. Note that *kV* is a bounded matrix for any given *k* and hence, given *t* large enough, it holds that $Qt+mV$ is a rate matrix for any *m* between 0 and *k*.

This shows that, for *t* large enough, $\exp (Qt)$ is an embeddable CS Markov matrix with at least $k+1$ different CS Markov generators. Moreover, $\exp (Qt)$ and all its generators have no null entries by construction and they can therefore be perturbed as in Theorem 3.3 in Casanellas et al. (2020b) to obtain a positive measure subset of embeddable CS Markov matrices that have $k+1$ CS Markov generators. $\square$

#### Remark 4

The perturbation presented in Theorem 3.3 in Casanellas et al. (2020b) consists of small changes on the real and complex parts of the eigenvalues and eigenvectors of *M* other than the eigenvector $(1,\dots ,1)$ and its corresponding eigenvalue 1. If those changes are small enough then the resulting transition matrix and all its generators still satisfy the stochastic constraints of Markov/rate matrices.

#### Remark 5

Using the same notation as in the proposition above and given $C\in GL_2({\mathbb {C}})$, let us define

$$\begin{aligned} Q(C) = P \;diag(I_2,C)\;diag\left( 1, -7\pi , -4\pi -\frac{3\pi }{2}i, -4\pi +\frac{3\pi }{2}i\right) \;diag(I_2,C^{-1})\;P^{-1}. \end{aligned}$$

Since $Q(I_2)=Q$ is a CS rate matrix with no null entries, so is *Q*(*C*) for $C\in GL_2({\mathbb {C}})$ close enough to $I_2$. Moreover, by construction we have that $\exp (2tQ(C))=\exp (2tQ)$ for all $t\in {\mathbb {N}}$. Therefore, for $t\in {\mathbb {N}}$ we have that $\exp (2tQ)$ has uncountably many Markov generators (i.e. 2*tQ*(*C*) with *C* close to $I_2$) and all of them are CS matrices (Culver 1966, Corollary 1). It is worth noting that according to (Culver 1966, Corollary 1), if a matrix has uncountably many logarithms, then it necessarily has repeated real eigenvalues. Therefore, the subset of embeddable CS Markov matrices with uncountably many generators has measure zero within the set of all matrices.

## Volumes of $4\times 4$ CS Markov matrices

In this section, we compute the relative volumes of embeddable $4\times 4$ CS Markov matrices within some meaningful subsets of Markov matrices. The aim of this section is to describe how large the different sets of matrices are compared to each other.

Let $V^{Markov}_4$ be the set of all $4\times 4$ CS Markov matrices. We use the following description

$$\begin{aligned} V^{Markov}_4= & {} \{(b,c,d,e,g,h)^T\in {\mathbb {R}}^6: b,c,d,e,g,h\ge 0,1-b-c-d\ge 0,\\{} & {} \quad \quad 1-e-g-h\ge 0\}. \end{aligned}$$

More explicitly, we identify the $4\times 4$ CS Markov matrix

$$\begin{aligned} \begin{pmatrix} 1-b-c-d&{}b&{}c&{}d\\ e&{}1-e-g-h&{}g&{}h\\ h&{}g&{}1-e-g-h&{}e\\ d&{}c&{}b&{}1-b-c-d\\ \end{pmatrix} \end{aligned}$$

with a point $(b,c,d,e,g,h)\in V^{Markov}_4.$ Let $V_+$ be the set of all CS Markov matrices having real positive eigenvalues, where

$$\begin{aligned} \Delta =((1-e-2g-h)-(1-b-c-2d))^2+4(e-h)(b-c), \end{aligned}$$

is the discriminant of the matrix $M_2$ as stated in Sect. 3. We have $V_+\subseteq V^{Markov}_4$. More explicitly,

$$\begin{aligned} V_+&=\{(b,c,d,e,g,h)\in {\mathbb {R}}^6 : b,c,d,e,g,h\ge 0,\\&\qquad 1-b-c-d\ge 0,\qquad 1-e-g-h\ge 0,\qquad 1-b-c-e-h>0,\\&\qquad (2-b-c-2d-e-2g-h)+\Delta>0,\quad (2-b-c-2d-e-2g-h)-\Delta>0,\quad \Delta >0\}. \end{aligned}$$

Let $V_{em+}$ be the set of all embeddable $4\times 4$ CS Markov matrices with four distinct real positive eigenvalues. We have $V_{em+}\subseteq V_+$. Therefore, by Theorem 2,

$$\begin{aligned} V_{em+}&=\{(b,c,d,e,g,h)\in {\mathbb {R}}^6 : b,c,d,e,g,h\ge 0,\qquad 1-b-c-d\ge 0,\qquad 1-e-g-h\ge 0,\\&\qquad 1-b-c-e-h>0,\\&\quad (2-b-c-2d-e-2g-h)+\Delta>0,\quad (2-b-c-2d-e-2g-h)-\Delta>0 , \quad \Delta >0,\\&\qquad |\phi (0)|\le -\alpha _{1},\qquad |\varepsilon (0)|\le -\beta _{1},\qquad \delta (0)\le \beta _{1},\qquad \gamma (0)\le \alpha _{1} \}. \end{aligned}$$

Finally, we consider the following two biologically relevant subsets of $V^{Markov}_4$. Let $V_{\mathrm{{DLC}}}$ be the set of *diagonally largest in column* (DLC) Markov matrices, which is the subset of $V^{Markov}_4$ containing all CS Markov matrices such that the diagonal element is the largest element in each column. These matrices are related to matrix parameter identifiability in phylogenetics (Chang 1996). Secondly, we let $V_{\mathrm{{DD}}}$ be the set of *diagonally dominant* (DD) Markov matrices, which is the subset of $V^{Markov}_4$ matrices containing all CS Markov matrices such that in each row the diagonal element is at least the sum of all the other elements in the row. Biologically, the subspace $V_{\mathrm{{DD}}}$ consists of matrices with probability of not mutating at least as large as the probability of mutating. If a diagonally dominant matrix is embeddable, it has an identifiable rate matrix (Cuthbert 1972; James 1973). By the definition of each set, we have the inclusion $V_{\mathrm{{DD}}}\subseteq V_{\mathrm{{DLC}}}$.

### Remark 6

The sets $V_+$, $V_{em+}$, $V_{\mathrm{{DLC}}}$, $V_{\mathrm{{DD}}}$ that we consider in this section are all subsets of the set $V^{Markov}_4$ of all $4\times 4$ CS Markov matrices, but we can use the same definition to refer to the equivalent subsets of $n\times n$ CS Markov matrices. Therefore, we will use the same notation $V_+$, $V_{em+}$, $V_{\mathrm{{DLC}}}$, $V_{\mathrm{{DD}}}$ to refer to the equivalent subsets of the set $V^{Markov}_n$ of $n\times n$ CS Markov matrices without confusion in the following sections.

In the rest of this section, the number *v*(*A*) denotes the Euclidean volume of the set *A*. By definition, $V^{Markov}_4$, $V_{\mathrm{{DLC}}}$ and $V_{\mathrm{{DD}}}$ are polytopes, since they are defined by the linear inequalities in ${\mathbb {R}}^6$. Hence, we can use Polymake (Gawrilow and Joswig 2000) to compute their exact volumes and obtain that

$$\begin{aligned} v(V^{Markov}_4)=\frac{1}{36},\quad v(V_{\mathrm{{DLC}}})= \frac{1}{576}\quad \text{ and } \quad v(V_{\mathrm{{DD}}})=\frac{1}{2304}. \end{aligned}$$

Hence, we see that $V_{\mathrm{{DLC}}}$ and $V_{\mathrm{{DD}}}$ constitute roughly only $6.25\%$ and $1.56\%$ of $V_4^{Markov}$, respectively.

On the other hand, we will estimate the volume of the sets $V_+, V_{em+}, V_{\mathrm{{DLC}}}\cap V_+,V_{\mathrm{{DLC}}}\cap V_{em+},V_{\mathrm{{DD}}}\cap V_+, \text{ and } V_{\mathrm{{DD}}}\cap V_{em+}$ using the hit-and-miss Monte Carlo integration method (Hammersley 2013) with sufficiently many sample points in Mathematica (Inc. 2022). Theoretically, Theorem 2 enables us to compute the exact volume of these relevant sets. For example in the case of K3P matrices, such exact computation of volumes has been feasible in Roca-Lacostena and Fernández-Sánchez (2018b). However, while for the K3P matrices, the embeddability criterion is given by three quadratic polynomial inequalities, in the case of CS matrices the presence of nonlinear and nonpolynomial constraints imposed on each set, makes the exact computation of the volume of these sets intractable. Therefore, we need to approximate the volume of these sets. Given a subset $A\subseteq V_4^{Markov}$, the volume estimate of *v*(*A*) computed using the hit-and-miss Monte Carlo integration method with *n* sample points is given by the number of points belonging to *A* out of *n* sample points. For computational purposes, in the formula of $\phi (0)$ and $\varepsilon (0)$, we use the fact that

$$\begin{aligned} y-z&=\log \left( \frac{(2-b-c-2d-e-2g-h)+\sqrt{\Delta }}{(2-b-c-2d-e-2g-h)-\sqrt{\Delta }}\right) .\\&=\log \left( \frac{((2-b-c-2d-e-2g-h)+\sqrt{\Delta })^2}{(2-b-c-2d-e-2g-h)^2-\Delta }\right) .\\&=\log \left( \frac{((2-b-c-2d-e-2g-h)+\sqrt{(b+c+2d-e-2g-h)^2+4(e-h)(b-c)})^2}{(2-b-c-2d-e-2g-h)^2-((b+c+2d-e-2g-h)^2+4(e-h)(b-c))}\right) \end{aligned}$$

All codes for the computations implemented Mathematica and Polymake can be found at the following address: https://github.com/ardiyam1/Embeddability-and-rate-identifiability-of-centrosymmetric-matrices.

The results of these estimations using the hit-and-miss Monte Carlo integration implemented in Mathematica with *n* sample points are presented in Table 1, while Table 2 provides an estimated volume ratio between relevant subsets of centrosymmetric Markov matrices using again the hit-and-miss Monte Carlo integration with *n* sample points. In Table 1, we firstly generate *n* centrosymmetric matrices whose off-diagonal entries were sampled uniformly in [0, 1] and forced the rows of the matrix to sum to one. Out of these *n* matrices, we test how many of them are actually Markov matrices (i.e. the diagonal entries are non-negative) and then out of these how many have positive eigenvalues. In particular, for $n=10^7$ sample points containing 277628 centrosymmetric Markov matrices, Table 2 suggests that there are approximately $1.7\%$ of centrosymmetric Markov matrices with distinct positive eigenvalues that are embeddable. Moreover, we can see that for $n=10^7$, out of all embeddable centrosymmetric Markov matrices with distinct positive eigenvalues, almost all are diagonally largest in column, while only $28\%$ are diagonally dominant.

**Table 1:** Number of samples in the sets

*n*	$10^4$	$10^5$	$10^6$	$10^7$
Samples in $V_4^{Markov}$	280	2767	27829	277628
Samples $V_+$	23	192	1999	20601
Samples in $V_{em+}$	3	34	359	3511
Samples in $V_{\mathrm{{DLC}}}\cap V_+$	19	154	1541	15830
Samples in $V_{\mathrm{{DD}}}\cap V_+$	3	31	262	2889
Samples in $V_{\mathrm{{DLC}}}\cap V_{em+}$	3	34	357	3503
Samples in $V_{\mathrm{{DD}}}\cap V_{em+}$	1	15	105	1011

$V_+, V_{em+}, V_{\mathrm{{DLC}}}\cap V_+,V_{\mathrm{{DLC}}}\cap V_{em+},V_{\mathrm{{DD}}}\cap V_+ \text{ and } V_{\mathrm{{DD}}}\cap V_{em+}$ using hit-and-miss methods and Theorem 2.

**Table 2:** Relative volumes ratio between the relevant subsets obtained using hit-and-miss method and Theorem 2. The volumes were estimated as the quotient of the sample sizes in Table 1

*n*	$10^4$	$10^5$	$10^6$	$10^7$
$\frac{v(V_{em+})}{v(V_+)}$	0.130435	0.177083	0.17959	0.170429
$\frac{v(V_{\mathrm{{DLC}}}\cap V_{em+})}{v(V_{\mathrm{{DLC}}}\cap V_+)}$	0.157895	0.220779	0.231668	0.221289
$\frac{v(V_{\mathrm{{DLC}}}\cap V_{em+})}{v(V_+)}$	0.130435	0.177083	0.178589	0.17004
$\frac{v(V_{\mathrm{{DLC}}}\cap V_{em+})}{v(V_{em+})}$	1	1	0.994429	0.997721
$\frac{v(V_{\mathrm{{DD}}}\cap V_{em+})}{v(V_{\mathrm{{DD}}}\cap V_+)}$	0.333333	0.483871	0.400763	0.349948
$\frac{v(V_{\mathrm{{DD}}}\cap V_{em+})}{v(V_+)}$	0.0434783	0.078125	0.052563	0.0490753
$\frac{v(V_{\mathrm{{DD}}}\cap V_{em+})}{v(V_{em+})}$	0.333333	0.441176	0.292479	0.287952

An alternative approach for approximating the number of embeddable matrices within the model is to use Algorithm 5.8 in Casanellas et al. (2023) to test the embeddability of the sample points. Tables  4 and 5 below are analogous to Tables 1 and 2, but Table 4 was obtained using the sampling method in (Roca-Lacostena 2021, Appendix A), while using either Algorithm 5.8 in Casanellas et al. (2023) or the inequalities in Theorem 2 yields identical results which are provided in Tables 4 and 5.

We used the python implementation of Algorithm 5.8 in Casanellas et al. (2023) provided in (Roca-Lacostena 2021, Appendix A) and modified it to sample on the set of $4 \times 4$ CS Markov matrices with positive eigenvalues. The original sampling method used in (Roca-Lacostena 2021, Appendix A) consisted of sampling uniformly on the set of $4\times 4$ centrosymmetric-Markov matrices until we obtained *n* samples (or as many samples as we require) with positive eigenvalues.

Despite the fact that Theorem 2 and Algorithm 5.8 in Casanellas et al. (2023) were originally implemented using different programming languages (Wolfram Mathematica and Python respectively) and were tested with different sample sets, the results obtained are quite similar as illustrated by Tables 2 and 5. In fact, when we apply both Algorithm 5.8 in Casanellas et al. (2023) and Theorem 2 on the same sample set in Table 3, we obtain identical results which are displayed in Tables 4 and 5.

**Table 3:** Number of samples in $V_+, V_{\mathrm{{DLC}}}\cap V_+, \text{ and } V_{\mathrm{{DD}}}\cap V_+$ obtained by using the sampling method in (Roca-Lacostena 2021, Appendix A)

Samples in $V_+$	$10^4$	$10^5$	$10^6$	$10^7$
Samples in $V_{\mathrm{{DLC}}}\cap V_+$	8531	85446	854709	8549100
Samples in $V_{\mathrm{{DD}}}\cap V_+$	1464	14538	144546	1448720

**Table 4:** Number of samples in $V_{em+}, \ V_{\mathrm{{DLC}}}\cap V_{em+} \text{ and } V_{\mathrm{{DD}}}\cap V_{em+}$ obtained by applying either Theorem 2 or the results in Casanellas et al. (2023) on the sample set in Table 3

Samples in $V_{em+}$	1877	18663	185357	1862413
Samples in $V_{\mathrm{{DLC}}}\cap V_{em+}$	1869	18586	184555	1854592
Samples in $V_{\mathrm{{DD}}}\cap V_{em+}$	516	5164	50058	504304

**Table 5:** Relative volumes ratio between the relevant subsets obtained using hit-and-miss method and either Algorithm 5.8 in Casanellas et al. (2023) or Theorem 2. The volumes were estimated as the quotient of the sample sizes in Tables 3 and 4

*n*	$10^4$	$10^5$	$10^6$	$10^7$
$\frac{v(V_{em+})}{v(V_+)}$	0.1877	0.18663	0.185357	0.1862413
$\frac{v(V_{\mathrm{{DLC}}}\cap V_{em+})}{v(V_{\mathrm{{DLC}}}\cap V_+)}$	0.2191	0.2175	0.2159	0.2169
$\frac{v(V_{\mathrm{{DLC}}}\cap V_{em+})}{v(V_+)}$	0.1869	0.18586	0.184555	0.1854592
$\frac{v(V_{\mathrm{{DLC}}}\cap V_{em+})}{v(V_{em+})}$	0.9957	0.9959	0.99567	0.99580
$\frac{v(V_{\mathrm{{DD}}}\cap V_{em+})}{v(V_{\mathrm{{DD}}}\cap V_+)}$	0.3524	0.3552	0.3463	0.3481
$\frac{v(V_{\mathrm{{DD}}}\cap V_{em+})}{v(V_+)}$	0.0516	0.05164	0.050058	0.0504
$\frac{v(V_{\mathrm{{DD}}}\cap V_{em+})}{v(V_{em+})}$	0.2749	0.2767	0.2701	0.2708

It is worth noting that the embeddability criteria given in Theorem 2 use inequalities depending on the entries of the matrix, whereas Algorithm 5.8 in Casanellas et al. (2023) relies on the computation of its principal logarithm and its eigenvalues and eigenvector, which may cause numerical issues when working with matrices with determinant close to 0. Moreover, the computation of logarithms can be computationally expensive. As a consequence, the algorithm implementing the criterion for embeddability arising from Theorem 2 is faster. Table 6 shows the running times for the implementation of both embeddability criteria used to obtain Table 5.

**Table 6:** Running times for the Python implementation of the embeddability criterion arising from Theorem 2 and from Algorithm 5.8 in Casanellas et al. (2023). The simulations were run using a computer with 8GB of memory

	$10^4$	$10^5$	$10^6$	$10^7$
Sampling time	12.5s	121.5s (2 min)	1222s (20min)	12141.8s (3h 22min)
Embedding criteria (Theorem 2)	28.3s	273.2s (4min 30s)	2703s (45min)	27413s (7h 37min)
Embedding criteria (Algorithm 5.8)	84.2s	840.5s (15 min)	8358 (2h 19min)	83786s (23h 16min)

The Python implementation of Algorithm 5.8 in Casanellas et al. (2023) provided in (Roca-Lacostena 2021, Appendix A) can also be used to test the embeddability of any $4\times 4$ CS Markov matrix (including those with non-real eigenvalues) without modifying the embeddability criteria. To do so, it is enough to apply the algorithm to a set of Markov matrices with different eigenvalues sampled uniformly from the set of all $4\times 4$ CS Markov matrix. As hinted in Remark 3, this would also be possible using the embedability criterion in Theorem 2 together with the boundaries for *k* provided in (Casanellas et al. 2023, Theorem 5.5). Table 7 shows the results obtained when applying Algorithm 5.8 in Casanellas et al. (2023) to a set of $10^7$ $4\times 4$ CS Markov matrices sampled uniformly.

**Table 7:** Embeddable matrices within $4\times 4$ CS Markov matrices and its intersection with DLC matrices and DD matrices

	Samples	Embeddable samples	Proportion of embeddable
$V_4^{Markov}$	$10^7$	173455	0.0173455
$V_{\mathrm{{DLC}}}$	1021195	172380	0.1688022
$V_{\mathrm{{DD}}}$	156637	49471	0.3158321

As most DLC and DD matrices have positive eigenvalues, the proportion of embeddable matrices within these subsets is almost the same when admitting matrices with non-positive eigenvalues (as in Table 7 instead of only considering matrices with positive eigenvalues as we did in Tables 2 and 5. On the other hand, the proportion of $4\times 4$ embeddable CS matrices is much smaller in this case.

## Centrosymmetric matrices and generalized Fourier transformation

In Sects. 3 and 4 we have seen the embeddability criteria for $4\times 4$ centrosymmetric Markov matrices and the volume of their relevant subsets. In this section, we are extending this framework to larger matrices. The importance of this extension is relevant to the goal of synthetic biology which aims to expand the genetic alphabet. For several decades, scientists have been cultivating ways to create novel forms of life with basic biochemical components and properties far removed from anything found in nature. In particular, they are working to expand the number of amino acids which is only possible if they are able to expand the genetic alphabet (see for example (Hoshika et al. 2019)).

### Properties of centrosymmetric matrices

For a fixed $n\in {\mathbb {N}}$, let $V_n$ denote the set of all centrosymmetric matrices of order *n*. Moreover, let $V_n^{Markov}$ and $V_n^{rate}$ denote the set of all centrosymmetric Markov and rate matrices of order *n*, respectively. As a subspace of the set of all $n\times n$ real matrices, for *n* even, dim$(V_n)=\frac{n^2}{2}$ while for *n* odd, dim$(V_n)=\lfloor \frac{n}{2}\rfloor (n+1)+1$. We will now mention some geometric properties of the sets $V_n^{Markov}$ and $V_n^{rate}.$ Furthermore, for any real number *x*, $\lfloor x\rfloor$ and $\lceil x\rceil$ denote the floor and the ceiling function of *x*, respectively.

#### Proposition 4

1. For *n* even, $V_n^{Markov}\subseteq {\mathbb {R}}^{\frac{n(n-1)}{2}}_{\ge 0}$ is a Cartesian product of $\frac{n}{2}$ standard $(n-1)$-simplices and its volume is $\frac{1}{(n-1)!^{\frac{n}{2}}}.$ For *n* odd, $V_n^{Markov}\subseteq {\mathbb {R}}^{\lfloor \frac{n}{2}\rfloor n}_{\ge 0}$ is a Cartesian product of $\lfloor \frac{n}{2}\rfloor$ standard $(n-1)$-simplices and the $\lfloor \frac{n}{2}\rfloor$-simplex with vertices $\{0, \frac{e_i}{2}\}_{1\le i\le \lfloor \frac{n}{2}\rfloor }\cup \{e_{\lfloor \frac{n}{2}\rfloor +1}\}$, where $e_i$ is the *i*-th standard unit vector in ${\mathbb {R}}^n$. Hence, the volume of $V_n^{Markov}$ is $\frac{1}{2^{\lfloor \frac{n}{2}\rfloor }(\lfloor \frac{n}{2}\rfloor )!(n-1)!^{\lfloor \frac{n}{2}\rfloor }}$.
2. For *n* even, $V_n^{rate}={\mathbb {R}}_{\ge 0}^{\frac{n(n-1)}{2}}$ and for *n* odd, $V_n^{rate}={\mathbb {R}}_{\ge 0}^{\lfloor \frac{n}{2}\rfloor n}$.

#### Proof

Here we consider the following identification for an $n\times n$ centrosymmetric matrix *M*. For *n* even, *M* can be thought as a point $(M_1,\dots , M_{\frac{n}{2}})\in ({\mathbb {R}}^n_{\ge 0})^{\frac{n}{2}}$ where the point $M_i\in {\mathbb {R}}^n_{\ge 0}$ corresponds to the *i*-th row of *M*. Similarly, for *n* odd, we identify *M* as a point in $({\mathbb {R}}^n_{\ge 0})^{\lfloor \frac{n}{2}\rfloor }\times {\mathbb {R}}^{\lfloor \frac{n}{2}\rfloor +1}_{\ge 0}$. Since *M* is a Markov matrix, under this identification, each point $M_i$ lies in some simplices. Therefore, $V^{Markov}_n$ is a Cartesian product of some simplices. For *n* even, these simplices are the standard $(n-1)$-dimensional simplex:

5.1 $$\begin{aligned} \left\{ \begin{array}{ll} x_1+\cdots +x_n=1,\\ x_i\ge 0,\quad 1\le i\le n \end{array} \right. \quad \Leftrightarrow \quad \left\{ \begin{array}{ll} x_1+\cdots +x_{n-1}\le 1,\\ x_i\ge 0,\quad 1\le i\le n-1 \end{array} \right. \end{aligned}$$

For *n* odd and $1\le i\le \lfloor \frac{n}{2}\rfloor$, the point $M_i$ belongs to standard $(n-1)$-simplex above and the point $M_{\lfloor \frac{n}{2}\rfloor +1}$ belongs to the simplex

5.2 $$\begin{aligned} \left\{ \begin{array}{ll} 2x_1+\cdots +2x_{\lfloor \frac{n}{2}\rfloor }+x_{\lfloor \frac{n}{2}\rfloor +1}=1\\ x_i\ge 0,\quad 1\le i\le \lfloor \frac{n}{2}\rfloor +1 \end{array} \right. \quad \Leftrightarrow \quad \left\{ \begin{array}{ll} x_1+\cdots +x_{\lfloor \frac{n}{2}\rfloor }\le \frac{1}{2},\\ x_i\ge 0,\quad 1\le i\le \lfloor \frac{n}{2}\rfloor \end{array} \right. \end{aligned}$$

We now compute the volume of $V^{Markov}_n$. Let us recall the fact that the volume of the Cartesian product of spaces is equal to the product of volumes of each factor space if the volume of each factor space is bounded. Moreover, the $(n-1)$-dimensional volume of the standard simplex in Eq. (5.1) in ${\mathbb {R}}^{n-1}$ is $\frac{1}{(n-1)!}.$ For *n* even, the statement follows immediately. For *n* odd, we use the fact that the $\lfloor \frac{n}{2}\rfloor$-dimensional volume of the simplex in Eq. (5.2) is $\frac{1}{2^{\lfloor \frac{n}{2}\rfloor }(\lfloor \frac{n}{2}\rfloor )!}.$ We refer the reader to Stein (1966) for an introductory text on the volume of simplices.

For the second statement, we use the fact that if *Q* is a rate matrix, then $q_{ii}=-\sum _{j\ne i}q_{ij}$ where $q_{ij}\ge 0$ for $i\ne j$. $\square$

In the rest of this section, let $J_n$ be the $n\times n$ anti-diagonal matrix, i.e. the (*i*, *j*)-entries are one if $i+j= n+1$ and zero otherwise. The following proposition provides some properties of the matrix $J_n$ that can be checked easily.

#### Proposition 5

Let $$A=(a_{ij})\in M_n({\mathbb {R}})$$. Then

1. $$(AJ_n)_{ij}=a_{i,n+1-j} \text{ and } (J_nA)_{ij}=a_{n+1-i,j}.$$
2. *A* is a centrosymmetric matrix if only if $J_nAJ_n=A.$

In Sect. 3, we have seen that $4\times 4$ CS matrices can be block-diagonalized through the matrix *S*. Now we will present a construction of generalized Fourier matrices to block-diagonalize any centrosymmetric matrices. Let us consider the following recursive construction of the $n\times n$ matrix $S_n$:

5.3 $$\begin{aligned} S_1=\begin{pmatrix} 1\\ \end{pmatrix}, S_2=\begin{pmatrix} 1&{}1\\ 1&{}-1\\ \end{pmatrix} \text{ and } S_n:=\begin{pmatrix} 1&{}0&{}1\\ 0&{}S_{n-2}&{}0\\ 1&{}0&{}-1\\ \end{pmatrix}, \text{ for } n\ge 3. \end{aligned}$$

#### Proposition 6

For each natural number $n\ge 3$, $S_n$ is invertible and its inverse is given by

$$\begin{aligned} S_n^{-1}=\begin{pmatrix} \frac{1}{2}&{}0&{} \frac{1}{2}\\ 0&{}S_{n-2}^{-1}&{}0\\ \frac{1}{2}&{}0&{} -\frac{1}{2}\\ \end{pmatrix}. \end{aligned}$$

#### Proof

The proposition easily follows from the definition of $S_n$. Indeed, we have

$$\begin{aligned} \begin{pmatrix} \frac{1}{2}&{}0&{} \frac{1}{2}\\ 0&{}S_{n-2}^{-1}&{}0\\ \frac{1}{2}&{}0&{} -\frac{1}{2}\\ \end{pmatrix}S_n=\begin{pmatrix} \frac{1}{2}&{}0&{} \frac{1}{2}\\ 0&{}S_{n-2}^{-1}&{}0\\ \frac{1}{2}&{}0&{} -\frac{1}{2}\\ \end{pmatrix}\begin{pmatrix} 1&{}0&{}1\\ 0&{}S_{n-2}&{}0\\ 1&{}0&{}-1\\ \end{pmatrix}=I_n. \end{aligned}$$

$$\square $$

The following proposition provides another block decomposition of the matrix $S_n$ and its inverse.

#### Proposition 7

Let $$n\ge 2.$$

1. For *n* even, $$S_n=\begin{pmatrix} I_{\frac{n}{2}}&{}J_{\frac{n}{2}}\\ J_{\frac{n}{2}}&{}-I_{\frac{n}{2}}\\ \end{pmatrix}$$, while for *n* odd, $$S_n=\begin{pmatrix} I_{\lfloor \frac{n}{2}\rfloor }&{}0&{}J_{\lfloor \frac{n}{2}\rfloor }\\ 0&{}1&{}0\\ J_{\lfloor \frac{n}{2}\rfloor }&{}0&{}-I_{\lfloor \frac{n}{2}\rfloor }\\ \end{pmatrix}.$$
2. Using these block partitions, $S_n^{-1}=\frac{1}{2}S_n$ for *n* even, while $$S_n^{-1}=\begin{pmatrix} \frac{1}{2}I_{\lfloor \frac{n}{2}\rfloor }&{}0&{} \frac{1}{2}J_{\lfloor \frac{n}{2}\rfloor }\\ 0&{}1&{}0\\ \frac{1}{2}J_{\lfloor \frac{n}{2}\rfloor }&{}0&{}- \frac{1}{2}I_{\lfloor \frac{n}{2}\rfloor }\\ \end{pmatrix}$$ for *n* odd.

#### Proof

The proof follows from induction on *n* and the fact that $J_n^2=I_n$. $\square$

We will call a vector $v\in {\mathbb {R}}^n$ *symmetric* if $v_i=v_{n+1-i}$ for every $1\le i\le n$, i.e. $J_n v=v.$ Moreover, we call a vector $w\in {\mathbb {R}}^n$ *anti-symmetric* if $v_i=-v_{n+1-i}$ for every $1\le i\le n$, i.e. $J_n v=-v.$ The following technical proposition will be used in what follows in order to simplify a centrosymmetric matrix.

#### Proposition 8

Let $n\ge 2$. Let $v\in {\mathbb {R}}^n$ be a symmetric vector and $w\in {\mathbb {R}}^n$ be an anti-symmetric vector.

1. The last $\lfloor \frac{n}{2}\rfloor$ entries of $S_nv$ and $v^TS_n$ are zero. Similarly, the last $\lfloor \frac{n}{2}\rfloor$ entries of $S_n^{-1}v$ and $v^TS_n^{-1}$ are zero.
2. The first $\lfloor \frac{n}{2}\rfloor$ entries of $S_nw$ and $w^TS_n$ are zero. Similarly, the first $\lfloor \frac{n}{2}\rfloor$ entries of $S_n^{-1}w$ and $w^TS_n^{-1}$ are zero.
3. Then the sum of the entries of $S_nv$ and $v^TS_n$ is the sum of the entries of *v*.
4. Then the sum of the entries of $S_n^{-1}v$ and $v^TS_n^{-1}$ is the sum of the first $\lceil \frac{n}{2}\rceil$ entries of *v*.

#### Proof

We will only prove the first part of item (1) in the proposition using mathematical induction on *n*. The base case for $n=2$ can be easily obtained. Suppose now that the proposition holds for all $k<n.$ Let $$v=\begin{pmatrix}v_1\\ v'\\ v_1 \end{pmatrix}\in {\mathbb {R}}^n$$ be a symmetric element. Then $v'\in {\mathbb {R}}^{n-2}$ is also symmetric. By direct computation we obtain

$$\begin{aligned} S_nv=\begin{pmatrix} 1&{}0&{}1\\ 0&{}S_{n-2}&{}0\\ 1&{}0&{}-1\\ \end{pmatrix}\begin{pmatrix} v_1\\ v'\\ v_1\\ \end{pmatrix}=\begin{pmatrix} 2v_1\\ S_{n-2}v'\\ 0\\ \end{pmatrix}. \end{aligned}$$

The last $\lfloor \frac{n-2}{2}\rfloor$ entries of $S_{n-2}v'$ are zero. Thus, the last $\lfloor \frac{n-2}{2}\rfloor +1=\lfloor \frac{n}{2}\rfloor$ entries of $S_nv$ are zero as well. The proof of the other statements can be obtained analogously using induction. In particular, let us note that the proof given for item (1) directly implies item (3). $\square$

For a fixed number *n*, let us define the following map:

$$\begin{aligned} F_n:&M_n({\mathbb {R}})&\rightarrow M_n({\mathbb {R}})\\&A&\mapsto F_n(A):=S_n^{-1}AS_n. \end{aligned}$$

For $n=4,$ we have seen that if *A* is a CS matrix, then $F_4(A)$ is a block-diagonal matrix where each block is of size $2\times 2$ and is given by $A_1$ and $A_2$. Moreover, the upper block is a Markov matrix. The following lemma provides a generalization to these results.

#### Lemma 5

Let $n\ge 2.$ Given an $n\times n$ CS matrix *A*,  $F_n(A)$ is the following block-diagonal matrix

$$\begin{aligned} F_n(A)=diag (A_1,A_2), \end{aligned}$$

where $A_1$ is a matrix of size $\lceil \frac{n}{2}\rceil \times \lceil \frac{n}{2}\rceil$. Furthermore, if *A* is a Markov (rate) matrix, then $A_1$ is also a Markov (rate) matrix.

#### Proof

First suppose that *n* is even. By (Cantoni and Butler 1976, Lemma 2), we can partition *A* into the following block matrices:

$$\begin{aligned} A=\begin{pmatrix} B_1&{}B_2\\ J_{\frac{n}{2}}B_2J_{\frac{n}{2}}&{}J_{\frac{n}{2}}B_1J_{\frac{n}{2}}\\ \end{pmatrix}, \end{aligned}$$

where $B_1$ and $B_2$ are of size $\lfloor \frac{n}{2}\rfloor \times \lfloor \frac{n}{2}\rfloor .$ By Proposition 7, we have

$$\begin{aligned} S_n^{-1}AS_n&=\frac{1}{2}\begin{pmatrix} I_{\frac{n}{2}}&{}J_{\frac{n}{2}}\\ J_{\frac{n}{2}}&{}-I_{\frac{n}{2}}\\ \end{pmatrix} \begin{pmatrix} B_1&{}B_2\\ J_{\frac{n}{2}}B_2J_{ \frac{n}{2}}&{}J_{\frac{n}{2}}B_1J_{ \frac{n}{2}}\\ \end{pmatrix} \begin{pmatrix} I_{\frac{n}{2}}&{}J_{\frac{n}{2}}\\ J_{\frac{n}{2}}&{}-I_{\frac{n}{2}}\\ \end{pmatrix}\\&=\begin{pmatrix} B_1+B_2J_{\frac{n}{2}}&{}0\\ 0&{}J_{\frac{n}{2}}B_1J_{\frac{n}{2}}-J_{\frac{n}{2}}B_2\\ \end{pmatrix}.\\ \end{aligned}$$

Choose $A_1=B_1+B_2J_{\frac{n}{2}}$. Now suppose that *A* is a Markov matrix. This means that each row of *A* sums to 1 and *A* has non-negative entries. Therefore, for $1\le k\le \frac{n}{2}$, we have

$$\begin{aligned} \sum _{j=1}^{\frac{n}{2}}(A_1)_{kj}=\sum _{j=1}^{\frac{n}{2}}(B_1+B_2J_{\frac{n}{2}})_{kj}=\sum _{j=1}^{\frac{n}{2}}(a_{kj}+a_{k,\frac{n}{2}+j})=\sum _{j=1}^{n}a_{kj}=1 \end{aligned}$$

and for $$1\le j\le \frac{n}{2}$$, $$(B_1+B_2J_{\frac{n}{2}})_{kj}=a_{kj}+a_{k,\frac{n}{2}+j}\ge 0.$$

Now we consider the case when *n* is odd. Again by (Cantoni and Butler 1976, Lemma 2), we can partition *A* into the following block matrices:

$$\begin{aligned} A=\begin{pmatrix} B_1&{}p&{}B_2\\ q&{}r&{}q J_{\lfloor \frac{n}{2}\rfloor }\\ J_{\lfloor \frac{n}{2}\rfloor }B_2J_{\lfloor \frac{n}{2}\rfloor }&{}J_{\lfloor \frac{n}{2}\rfloor }p&{}J_{\lfloor \frac{n}{2}\rfloor }B_1J_{\lfloor \frac{n}{2}\rfloor }\\ \end{pmatrix}, \end{aligned}$$

where $B_1, B_2 \in M_{\lfloor \frac{n}{2}\rfloor \times \lfloor \frac{n}{2}\rfloor }({\mathbb {R}})$, $p \text{ and } q\in M_{1\times \lfloor \frac{n}{2}\rfloor }({\mathbb {R}})$ and $r\in M_{1 \times 1}({\mathbb {R}}).$ By Proposition 7, we have

$$\begin{aligned} S_n^{-1}AS_n&=\begin{pmatrix} \frac{1}{2}I_{\lfloor \frac{n}{2}\rfloor }&{}0&{} \frac{1}{2}J_{\lfloor \frac{n}{2}\rfloor }\\ 0&{}1&{}0\\ \frac{1}{2}J_{\lfloor \frac{n}{2}\rfloor }&{}0&{}- \frac{1}{2}I_{\lfloor \frac{n}{2}\rfloor }\\ \end{pmatrix}\begin{pmatrix} B_1&{}p&{}B_2\\ q&{}r&{}q J_{\lfloor \frac{n}{2}\rfloor }\\ J_{\lfloor \frac{n}{2}\rfloor }B_2J_{\lfloor \frac{n}{2}\rfloor }&{}J_{\lfloor \frac{n}{2}\rfloor }p&{}J_{\lfloor \frac{n}{2}\rfloor }B_1J_{\lfloor \frac{n}{2}\rfloor }\\ \end{pmatrix}\begin{pmatrix} I_{\lfloor \frac{n}{2}\rfloor }&{}0&{}J_{\lfloor \frac{n}{2}\rfloor }\\ 0&{}1&{}0\\ J_{\lfloor \frac{n}{2}\rfloor }&{}0&{}-I_{\lfloor \frac{n}{2}\rfloor }\\ \end{pmatrix}\\&=\begin{pmatrix} B_1+B_2J_{\lfloor \frac{n}{2}\rfloor }&{}p&{}0\\ 2q&{}r&{}0\\ 0&{}0&{}J_{\lfloor \frac{n}{2}\rfloor }B_1J_{\lfloor \frac{n}{2}\rfloor }-J_{\lfloor \frac{n}{2}\rfloor }B_2\\ \end{pmatrix}.\\ \end{aligned}$$

In this case, choose $$A_1=\begin{pmatrix} B_1+B_2J_{\lfloor \frac{n}{2}\rfloor }&{}p\\ 2q&{}r\\ \end{pmatrix}.$$ Suppose that *A* is a Markov matrix. Since each row of *A* sums to 1, we have

$$\begin{aligned} \sum _{j=1}^{\lfloor \frac{n}{2}\rfloor }2q_{1j}+r=\sum _{j=1}^{n}a_{\lfloor \frac{n}{2}\rfloor +1,j}=1 \end{aligned}$$

and for $$1\le k\le \lfloor \frac{n}{2}\rfloor $$,

$$\begin{aligned} \sum _{j=1}^{\lfloor \frac{n}{2}\rfloor }(B_1+B_2J_{\lfloor \frac{n}{2}\rfloor })_{kj}+p_{k1}=\sum _{j=1}^{\lfloor \frac{n}{2}\rfloor }(a_{kj}+a_{k,\lfloor \frac{n}{2}\rfloor +j+1})+a_{k,\lfloor \frac{n}{2}\rfloor +1}=\sum _{j=1}^{n}a_{kj}=1. \end{aligned}$$

From the fact that the entries of *A* are non-negative, for $1\le k, j\le \lfloor \frac{n}{2}\rfloor$, we obtain that

$$\begin{aligned} (B_1+B_2J_{\lfloor \frac{n}{2}\rfloor })_{kj}=a_{k,j}+a_{k, \lfloor \frac{n}{2}\rfloor +j}\ge 0. \end{aligned}$$

Therefore, all entries of $A_1$ sum to 1 and are non-negative meaning that $A_1$ is a Markov matrix as well. We can proceed similarly for the case when *A* is a rate matrix. $\square$

#### Lemma 6

For any natural number *n*, let $A_1=(\alpha _{i,j}),$ $A_2=(\beta _{i,j}) \in M_{\lceil \frac{n}{2}\rceil \times \lceil \frac{n}{2}\rceil }({\mathbb {R}})$. Suppose that $Q=diag(A_1,A_2)$ is a block diagonal matrix. Then

1. $F_n^{-1}(Q):=S_nQS_n^{-1}$ is a CS matrix.
2. $F_n^{-1}(Q)$ is a Markov matrix if and only if $A_1$ is a Markov matrix and for any $1\le i,j\le \lfloor \frac{n}{2}\rfloor ,$
  $$\begin{aligned} \alpha _{ij}+\beta _{\lfloor \frac{n}{2}\rfloor +1-i,\lfloor \frac{n}{2}\rfloor +1-j}\ge 0 \text{ and } \alpha _{i,\lfloor \frac{n}{2}\rfloor +1-j}-\beta _{\lfloor \frac{n}{2}\rfloor +1-i,j}\ge 0. \end{aligned}$$
3. $F_n^{-1}(Q)$ is a rate matrix if and only if $A_1$ is a rate matrix and for any $1\le i,j\le \lfloor \frac{n}{2}\rfloor ,$ such that for $i=j$, $\alpha _{ii}+\beta _{\lfloor \frac{n}{2}\rfloor +1-i,\lfloor \frac{n}{2}\rfloor +1-i}\le 0$ and for $i\ne j$,
  $$\begin{aligned} \alpha _{ij}+\beta _{\lfloor \frac{n}{2}\rfloor +1-i,\lfloor \frac{n}{2}\rfloor +1-j}\ge 0 \text{ and } \alpha _{i,\lfloor \frac{n}{2}\rfloor +1-j}-\beta _{\lfloor \frac{n}{2}\rfloor +1-i,j}\ge 0. \end{aligned}$$

#### Proof

We will only prove the lemma for *n* even. Similar arguments will work for *n* odd as well. By Proposition 7,

$$\begin{aligned} F_n^{-1}(Q)=\frac{1}{2} \begin{pmatrix} I_{\frac{n}{2}}&{}J_{\frac{n}{2}}\\ J_{\frac{n}{2}}&{}-I_{\frac{n}{2}}\\ \end{pmatrix} \begin{pmatrix} A_1&{}0\\ 0&{}A_2\\ \end{pmatrix} \begin{pmatrix} I_{\frac{n}{2}}&{}J_{\frac{n}{2}}\\ J_{\frac{n}{2}}&{}-I_{\frac{n}{2}}\\ \end{pmatrix}&=\frac{1}{2} \begin{pmatrix} A_1+J_{\frac{n}{2}}A_2J_{\frac{n}{2}}&{}A_1J_{\frac{n}{2}}-J_{\frac{n}{2}}A_2\\ J_{\frac{n}{2}}A_1-A_2J_{\frac{n}{2}}&{}J_{\frac{n}{2}}A_1J_{\frac{n}{2}}+A_2\\ \end{pmatrix}. \end{aligned}$$

Since $J_{\frac{n}{2}}(A_1+J_{\frac{n}{2}}A_2J_{\frac{n}{2}})J_{\frac{n}{2}}=J_{\frac{n}{2}}A_1J_{\frac{n}{2}}+A_2$ and $J_{\frac{n}{2}}(A_1J_{\frac{n}{2}}-J_{\frac{n}{2}}A_2)J_{\frac{n}{2}}= J_{\frac{n}{2}}A_1-A_2J_{\frac{n}{2}}$, then by (Cantoni and Butler 1976, Lemma 2), $F_n^{-1}(Q)$ is centrosymmetric which proves (1). For $1\le i\le \frac{n}{2}$,

$$\begin{aligned} \sum _{j=1}^n(F_n^{-1}(Q))_{ij}&=\frac{1}{2} \sum _{j=1}^n(\alpha _{i,j}+\beta _{\frac{n}{2}+1-i,\frac{n}{2}+1-j}+\alpha _{i,\frac{n}{2}+1-j}-\beta _{\frac{n}{2}+1-i,j})=\sum _{j=1}^n\alpha _{ij}. \end{aligned}$$

The above equality means that for $1\le i\le \frac{n}{2}$, the *i*-th row sum of $F_n^{-1}(Q)$ and $A_1$ coincide. This implies that if $F_n^{-1}(Q)$ is a Markov (rate) matrix, then $A_1$ is a Markov (rate) matrix as well. Additionally, note that

$$\begin{aligned} ( A_1+J_{\frac{n}{2}}A_2J_{\frac{n}{2}})_{ij}=\alpha _{i,j}+\beta _{\frac{n}{2}+1-i,\frac{n}{2}+1-j} \text{ and } (A_1J_{\frac{n}{2}}-J_{\frac{n}{2}}A_2)_{ij} =\alpha _{i,\frac{n}{2}+1-j}-\beta _{\frac{n}{2}+1-i,j}. \end{aligned}$$

Hence, (2) and (3) will follow immediately.$\square$

### Logarithms of centrosymmetric matrices

For the special structure encoded by the centrosymmetric matrices, one may ask whether they have logarithms which are also centrosymmetric. In this section, we provide some answers to this question.

#### Theorem 3

Let $A\in M_n({\mathbb {R}})$ be a CS matrix. Then *A* has a CS logarithm if and only if both the upper block matrix $A_1$ and the lower block matrix $A_2$ in Lemma 5 admit a logarithm.

#### Proof

Suppose that *A* has a centrosymmetric logarithm *Q*. By Lemma 5, $F_n(A)=\text{ diag }(A_1,A_2)$ and $F_n(Q)=\text{ diag }(Q_1,Q_2).$ Then $\exp (Q)=A$ implies that $\exp (Q_1)=A_1$ and $\exp (Q_2)=A_2.$ Hence, $A_1$ and $A_2$ admit a logarithm. Conversely, suppose that $A_1$ and $A_2$ admit a logarithm $Q_1$ and $Q_2$, respectively. Then the matrix $\text{ diag }(Q_1,Q_2)$ is a logarithm of the matrix $\text{ diag }(A_1,A_2)$. By Lemma 6, the matrix $F_n^{-1}(\text{ diag }(Q_1,Q_2))$ is a centrosymmetric logarithm of *A*. $\square$

#### Proposition 9

Let $A\in M_n({\mathbb {R}})$ be a CS matrix. If *A* is invertible, then it has infinitely many CS logarithms.

#### Proof

The assumptions imply that the matrices $A_1$ and $A_2$ in Lemma 5 are invertible. By (Higham 2008, Theorem 1.28), each $A_1$ and $A_2$ has infinitely many logarithms. Hence, Theorem 3 implies that *A* has infinitely many centrosymmetric logarithms. $\square$

#### Proposition 10

Let $A\in M_n({\mathbb {R}})$ be a CS matrix such that *Log*(*A*) is well-defined. Then *Log*(*A*) is again centrosymmetric.

#### Proof

Let us suppose that *Log*(*A*) is not centrosymmetric matrix. Define the matrix $Q=J_n(Log(A))J_n$. Then $Q\ne Log(A)$ since *Log*(*A*) is not centrosymmetric. It is also clear that $\exp (Q)=A$. Moreover, since $J_n^2=I_n$, the matrices *Log*(*A*) and *Q* have the same eigenvalues. Therefore, *Q* is also a principal logarithm of *A*, a contradiction to the uniqueness of principal logarithm. Hence, *Log*(*A*) must be centrosymmetric. $\square$

The following theorem characterizes the logarithms of any invertible CS Markov matrices.

#### Theorem 4

Let $A\in M_n({\mathbb {R}})$ be an invertible CS Markov matrix. Let $A_1=N_1D_1N_1^{-1}$ where $D_1=diag(R_1,R_2,\dots , R_l)$ is a Jordan form of the upper block matrix in Lemma 5. Similarly, let $A_2=N_2D_2N_2^{-1}$ where $D_2=diag(T_1,T_2,\dots , T_l)$ is a Jordan form of the lower block matrix in Lemma 5. Then *A* has a countable infinitely many logarithms given by

$$\begin{aligned} Q:=S_n N D N^{-1}S_n^{-1}, \end{aligned}$$

where

$$\begin{aligned} N:=diag (N_1,N_2)\quad \text{ and } \quad D:=diag (D_1',D_2'), \end{aligned}$$

and $D_i'$ denotes a logarithm of $D_i$. In particular, these logarithms of *A* are primary functions of *A*.

#### Proof

The theorem follows immediately from (Higham 2008, Theorem 1.28). $\square$

For the definition of primary function of a matrix, we refer the reader to Higham (2008). The above theorem says that the logarithms of a nonsingular centrosymmetric matrix contains a countable infinitely many primary logarithms and they are centrosymmetric matrices as well.

Finally, we will present a necessary condition for embeddability of CS Markov matrices in higher dimensions.

#### Lemma 7

Let $n\ge 2$. Suppose that $A=(a_{ij})$ is an embeddable CS Markov matrix of size $n\times n$ with a CS logarithm. Then for *n* even,

$$\begin{aligned} \sum _{j=1}^{\frac{n}{2}}(a_{jj}+a_{j,n-j+1})>1, \end{aligned}$$

while for *n* odd,

$$\begin{aligned} \sum _{j=1}^{\lfloor \frac{n}{2}\rfloor }(a_{jj}+a_{j,n-j+1})+a_{\lfloor \frac{n}{2}\rfloor +1,\lfloor \frac{n}{2}\rfloor +1}>1. \end{aligned}$$

#### Proof

Since *A* is an embeddable matrix with CS logarithm, we write $A=\exp (Q)$ for some CS rate matrix *Q*, and then

$$\begin{aligned} F_n(A)=F_n(\exp (Q))=\exp (F_n(Q)). \end{aligned}$$

By Lemma 5, for the centrosymmetric matrices *A*, *Q*, we have $F_n(A)=diag (A_1,A_2)$ and $F_n(Q)=diag (Q_1,Q_2)$ where $A_1$ is a Markov matrix and $Q_1$ is a rate matrix of size $\lceil \frac{n}{2}\rceil \times \lceil \frac{n}{2}\rceil .$ Therefore, $A_1=\exp (Q_1).$ If $\lambda _1,\cdots , \lambda _{\lceil \frac{n}{2}\rceil }$ are the eigenvalues, perhaps not distinct, of $Q_1,$ then the eigenvalues of $A_1$ are $e^{\lambda _1},\cdots ,e^{\lambda _{\lceil \frac{n}{2}\rceil }}$. Since one of $\lambda _i$’s is zero, then the trace of $A_1$ which is the sum of its eigenvalues is equal to

$$\begin{aligned} tr(A_1)=\sum _{j=1}^{\lceil \frac{n}{2}\rceil }e^{\lambda _j}>1. \end{aligned}$$

We now need to show that trace of $A_1$ has the form written in the lemma. Suppose that *n* is even. By the proof of Lemma 5, then

$$\begin{aligned} tr(A_1)=\sum _{j=1}^{\frac{n}{2}}(B_1+B_2J_{\frac{n}{2}})_{jj}=\sum _{j=1}^{\frac{n}{2}}(a_{jj}+a_{j,\frac{n}{2}+j})=\sum _{j=1}^{\frac{n}{2}}(a_{jj}+a_{j,n-j+1}). \end{aligned}$$

The proof for odd *n* can be obtained similarly.$\square$

Let $X_n\subseteq V_n^{Markov}$ be the subset containing all centrosymmetric-embeddable Markov matrices. We want to obtain an upper bound of the volume of $X_n$ using Lemma 7. Let $Y_n\subseteq V_n^{Markov}$ be the subset containing all centrosymmetric Markov matrices such that after applying the generalized Fourier transformation, the trace of the upper block matrix is greater than 1. The previous lemma implies that $X_n\subseteq Y_n$ and hence, $v(X_n)\le v(Y_n)$. Moreover, the upper bound $v(Y_n)$ is easy to compute as $Y_n$ is a polytope and for some values of *n*, these volumes are presented in Table 8. We see from Table 8, there are at most $50\%$ of matrices in $V_4^{Markov}$ that are centrosymmetically-embeddable and hence this upper bound $v(Y_4)$ is not good. For $n=5$, approximately, there are at most $62\%$ in $V_4^{Markov}$ that are centrosymmetrically-embeddable but for $n=6$, this upper bound gives a better proportion, which is approximately $0.1\%$.

**Table 8:** The exact volume $v(Y_n), n\in \{4,5,6\}$ computed using Polymake

	Dimension of $V_n^{Markov}$	$v(Y_n)$	$v(V_n^{Markov})$
$n=4$	6	$\frac{1}{72}\approx 1.39\times 10^{-2}$	$\frac{1}{36}\approx 2.78\times 10^{-2}$
$n=5$	10	$\frac{653}{4838400}\approx 1.35\times 10^{-4}$	$\frac{1}{4608}\approx 2.17\times 10^{-4}$
$n=6$	15	$\frac{433}{653837184000}\approx 6.22 \times 10^{-10}$	$\frac{1}{1728000}\approx 5.79\times 10^{-7}$

## Embeddability of $6\times 6$ centrosymmetric matrices

Throughout this section we shall consider *A* to be a $6 \times 6$ centrosymmetric Markov matrix with distinct eigenvalues. In particular, the matrices considered in this section are diagonalizable and are a dense subset of all $6 \times 6$ centrosymetric Markov matrices. Note that this notation differs from the notation for Markov matrices used in previous sections in order to make it consistent with the notation used in the results presented for generic centrosymmetric matrices.

In the previous section, we showed that *F*(*A*) is a block-diagonal real matrix composed of two $3\times 3$ blocks denoted by $A_1$ and $A_2$. Since both $A_1$ and $A_2$ have real entries, each of these matrices has at most one conjugate pair of eigenvalues. Adapting the notation introduced in Theorem 4 to diagonalizable matrices we have $N_1,N_2 \in GL_3({\mathbb {C}})$ such that $A_1=N_1 diag(1,\lambda _1, \lambda _2) N_1^{-1}$ and $A_2=N_2 diag(\mu ,\gamma _1, \gamma _2) N_2^{-1}$ with $\mu \in {\mathbb {R}}_{>0}$ and $\lambda _i,\gamma _i \in {\mathbb {C}}{\setminus } {\mathbb {R}}_{\ge 0}$. Moreover, we can assume that $Im(\lambda _1)>0$ without loss of generality (this can be achieved by permuting the second and third columns of $N_1$ if necessary). For ease of reading, we will define as $P:= S_6 diag(N_1,N_2)$, where $S_6$ is the matrix used to obtain the Fourier transform *F*(*A*) and was introduced in Sect. (5.3).

Next we give a criterion for the embeddability of *A* for each of the following cases: 
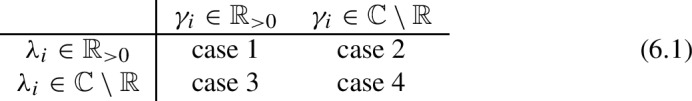


### Proposition 11

If a $6\times 6$ cetrosymmetric Markov matrix *A* does not belong to any of the cases in Table 6.1, then it is not embeddable.

### Proof

If *A* satifies the hypothesis of the proposition then either it has a null eigenvalue or it has a simple negative eigenvalue. In the former case *A* is a singular matrix and hence it has no logarithm. If *A* had a simple negative eigenvalue, then all its logarithms would have a non-real eigenvalue whose complementary pair is not an eigenvalue of *A* (otherwise *M* would have a repeated eigenvalues). Therefore, *A* has no real logarithm.

$$\square $$

### Remark 7

All the results in this section can be adapted to $5\times 5$ centrosymmetric Markov matrices by not considering the eigenvalue $\mu$ and modifying the forthcoming definitions of the matrices $Log_{-1}(A)$ and *V* accordingly (i.e. removing the fourth row and column in the corresponding diagonal matrix). In addition, these results still hold if the eigenvalue 1 of the Markov matrix has multiplicity 2.

### Case 1

The results for this case are not restricted to centrosymmetric matrices but can be applied to decide the embeddability of any suitable Markov matrix.

#### Proposition 12

If all the eigenvalues of a Markov matrix *A* are distinct and positive, then *A* is embeddable if and only if *Log*(*A*) is a rate matrix.

#### Proof

If *A* has distinct real eigenvalues then it has only one real logarithm, which is *Log*(*A*) (see (Culver 1966)). $\square$

### Case 2

In this case *A* has exactly one conjugate pair of complex eigenvalues and we obtain the following criterion by adapting Corollary 5.6 in Casanellas et al. (2023) to our framework:

#### Proposition 13

Given the matrix $V:= P \; diag(0,0,0,0,2\pi i, -2\pi i) \;P^{-1}$ define:and set $\ {\mathcal {N}}:= \{(i,j): i\ne j, \ V_{i,j}=0 \text { and } Log(A)_{i,j}<0\}.$ Then,

1. *A* is embeddable if and only if ${\mathcal {N}} = \emptyset$ and ${\mathcal {L}} \le {\mathcal {U}}$.
2. the set of Markov generators for *A* is $\Big \{ Q=Log(A) + k V: k\in {\mathbb {Z}} \text { such that } {\mathcal {L}} \le k \le {\mathcal {U}} \Big \}$.

#### Proof

The proof of this theorem is analogous to the proof of Theorem 5.5 in Casanellas et al. (2020a) but considering the matrix *V* as defined here. According to Proposition 1, any Markov generator of *A* is of the form

$$\begin{aligned} Log_k(A)&= P diag(0, \log (\lambda _1),\log (\lambda _2), \log (\mu ), \log _k(\gamma _1), \overline{\log _k(\gamma _1)}) P^{-1}\\&= P diag(0, \log (\lambda _1),\log (\lambda _2), \log (\mu ), \log _k(\gamma _1)+2\pi ki, \overline{\log _k(\gamma _1)}-2\pi k i) P^{-1}.\\ \end{aligned}$$

Such a logarithm can be rewritten as $Log(A)+kV$. Using this, we will prove that $Log_k(A)=Log(A)+kV$ is a rate matrix if and only if ${\mathcal {N}} = \emptyset$ and ${\mathcal {L}} \le k \le {\mathcal {U}}$.

Suppose that there exists $k\in {\mathbb {Z}}$ such that $Log_k(A)$ is a rate matrix. Hence, $Log(A)_{i,j}+kV_{i,j}\ge 0$ for all $i\ne j$. For $i\ne j$, we have:

- $Log(A)_{i,j}\ge 0$ for all $i\ne j$ such that $V_{i,j}=0$. This means that ${\mathcal {N}}=\emptyset .$
- $-\frac{Log(A)_{i,j}}{V_{i,j}}\le k$ for all $i\ne j$ such that $V_{i,j}>0$. This means that ${\mathcal {L}}\le k$.
- $-\frac{Log(A)_{i,j}}{V_{i,j}}\ge k$ for all $i\ne j$ such that $V_{i,j}<0$. This means that $k\le {\mathcal {U}}$.

Conversely, suppose that ${\mathcal {N}} = \emptyset$ and and that there is $k\in {\mathbb {Z}}$ such that ${\mathcal {L}} \le k \le {\mathcal {U}}$. We want to check that $Log_k(A)$ is a rate matrix. According to Proposition 1, each row of $Log_k(A)$ sums to 0. Moreover, for $i\ne j$, we have:

- if $V_{i,j}\!=\!0$, then $Log_k(A)_{i,j}\!=\!Log(A)_{i,j}$. Since ${\mathcal {N}}\!=\!\emptyset$, $Log_k(A)_{i,j}\!=\!Log(A)_{i,j}\ge 0$.
- if $$V_{i,j}\!>\!0$$, then $$Log_k(A)_{i,j}\!=\!Log(A)_{i,j}\!+\!k V_{i,j}\!\ge \! Log(A)_{i,j}\!+\!{\mathcal {L}} V_{i,j}\!\ge \! Log(A)_{i,j}+(-\frac{Log(A)_{i,j}}{V_{i,j}}) V_{i,j}=0.$$
- if $$V_{i,j}<0$$, then $$-Log_k(A)_{i,j}=-Log(A)_{i,j}-k V_{i,j}\le -Log(A)_{i,j}-{\mathcal {U}} V_{i,j}\le -Log(A)_{i,j}-(-\frac{Log(A)_{i,j}}{V_{i,j}}) V_{i,j}=0.$$

The proof is now complete. $\square$

### Case 3

As in Case 2, *A* has exactly one conjugate pair of eigenvalues and hence its embeddability (and all its generators) can be determined by using Proposition 13 but defining the matrix *V* as $V=P \; diag(0,0,0,0,2\pi i, -2\pi i) \;P^{-1}$. However in Case 3 the conjugate pair of eigenvalues lie in $A_1$ which is a Markov matrix. This allows us to use the results regarding the embeddability of $3\times 3$ Markov matrices to obtain an alternative criterion to test the embeddability of *A*. To this end we define

6.2 $$\begin{aligned} Log_{-1}(A):= P \; diag(0, z, {\overline{z}},\log (\mu ), \log (\gamma _1)\log (\gamma _2)) \;P^{-1} \end{aligned}$$

where $$z:= \log _{-1}(\lambda _1)$$.

#### Proposition 14

The matrix *A* is embeddable if and only if *Log*(*A*) or $Log_{-1}(A)$ are rate matrices.

#### Proof

Note that $\exp (Log(A))=\exp (Log_{-1}(A))=A$ so one of the implications is immediate to prove. To prove the other implication, we assume that *A* is embeddable and let *Q* be a Markov generator for it. Proposition 1 yields that

$$\begin{aligned} Q=P diag(0,\log _{k_1}(\lambda _1),\log _{k_2}(\lambda _2),\log _{k_3}(\mu ),\log _{k_4}(\gamma _1),\log _{k_5}(\gamma _2)) \; P^{-1}, \end{aligned}$$

for some integers $k_1,\dots ,k_5 \in {\mathbb {Z}}$. Therefore, $$F(Q)=\begin{pmatrix} Q_1 &{} 0\\ 0 &{} Q_2\\ \end{pmatrix}$$ where $Q_1$ and $Q_2$ are real logarithms of $A_1$ and $A_2$ respectively.

Since $A_2$ is a real matrix with distinct positive eigenvalues, its only real logarithm is its principal logarithm. This implies that $k_3=k_4=k_5=0$ (so that $Q_2=Log(A_2)$).

Now, recall that $A_1$ is a Markov matrix (see Lemma 5). Using Proposition 1 again, we obtain that $Q_1$ is a rate matrix, thus $A_1$ is embeddable. To conclude the proof it is enough to recall Theorem 4 in James (1973), which yields that $A_1$ is embeddable if and only if $Log(A_1)$ or $P_1\;diag(0,z,{\overline{z}}) \;P_1^{-1}$ is a rate matrix. $\square$

### Case 4

In this case, the solution to the embedding problem can be obtained as a byproduct of the results for the previous cases:

#### Proposition 15

Let $Log_{0,0}(A)$ denote the principal logarithm of *A* and $Log_{-1,0}(A)$ denote the matrix in (6.2). Given the matrix $V:= P \; diag(0,0,0,0,2\pi i, -2\pi i) \;P^{-1}$ and $k\in \{0,-1\}$ define:and set $\ {\mathcal {N}}_k:= \{(i,j): i\ne j, \ V_{i,j}=0 \text { and } Log_{k,0}(A)_{i,j}<0\}.$ Then,

1. *A* is embeddable if and only if ${\mathcal {N}}_k = \emptyset$ and ${\mathcal {L}}_k \le {\mathcal {U}}_k$ for $k=0$ or $k=-1$.
2. If *A* is embeddable, then at least one of its Markov generator can be written as
  $$\begin{aligned} Log_{k,k_2}(A):=P\; diag(0, \log _{k}(\lambda _1),\overline{\log _{k}(\lambda _1)}, \log (\mu ), \log _{k_2}(\gamma _1), \overline{\log _{k_2}(\gamma _1)}) \;P^{-1} \end{aligned}$$
  with $k\in \{0,-1\}$ and $k_2\in {\mathbb {Z}}$ such that ${\mathcal {L}}_{k} \le k_2 \le {\mathcal {U}}_{k}$.

#### Proof

The matrix *A* is embeddable if and only if it admits a Markov generator. According to Proposition 1, if such a generator *Q* exists then it can be written as $Log_{k_1,k_2}(A)$ for some $k_1,k_2 \in {\mathbb {Z}}$. Therefore, Lemma 5 implies that $$F(A)=\begin{pmatrix} A_1 &{} 0\\ 0 &{} A_2\\ \end{pmatrix}$$ for some matrices $A_1$ and $A_2$. Moreover, $$F(Q)=\begin{pmatrix} Q_1 &{} 0\\ 0 &{} Q_2\\ \end{pmatrix}$$ where $Q_1$ and $Q_2$ are real logarithms of $A_1$ and $A_2$ respectively.

As shown in the proof of Proposition 14, $A_1$ is actually a Markov matrix and $Q_1$ is a Markov generator for it (see also Lemma 5). Moreover, by Theorem 4 in James (1973), $A_1$ is embeddable if and only if $Log(A_1)$ or $Log_{-1}(A_1)$ are rate matrices. This implies that $Log_{k_1,k_2}(A)$ is a rate matrix if and only if $Log_{0,k_2}(A)$ or $Log_{-1,k_2}$ are rate matrices. To conclude the proof we proceed as in the proof of Proposition 13. Indeed, note that for $k\in \{0,-1\}$, $Log_{k,k_2}(A) = Log_{k,0}(A) + k_2 V$. Using this, it is immediate to check that $Log_{k,k_2}(A)$ is a rate matrix if and only if ${\mathcal {N}}_{k} = \emptyset$ and ${\mathcal {L}}_{k} \le k_2\le {\mathcal {U}}_{k}$. $\square$

## Discussion

The central symmetry is motivated by the complementarity between both strands of the DNA. When a nucleotide substitution occurs in one strand, there is also a substitution between the corresponding complementary nucleotides on the other strand. Therefore, working with centrosymmetric Markov matrices is the most general approach when considering both DNA strands.

In this paper, we have discussed the embedding problem for centrosymmetric Markov matrices. In Theorem 2, we have obtained a characterization of the embeddabilty of $4\times 4$ centrosymmetric Markov matrices which are exactly the strand symmetric Markov matrices. In particular, we have also shown that if a $4\times 4$ CS Markov matrix is embeddable, then any of its Markov generators is also a CS matrix. Furthermore, In Sect. 6, we have discussed the embeddability criteria for larger centrosymmetric matrices.

As a consequence of the characterization of Theorem 2, we have been able to compute and compare the volume of the embeddable $4\times 4$ CS Markov matrices within some subspaces of $4\times 4$ CS Markov matrices. These volume comparisons can be seen in Table 2 and Table 7. For larger matrices, using the results in Sect. 6, we have estimated the proportion of embeddable matrices within the set of all $6\times 6$ centrosymmetric Markov matrices and within the subsets of DLC and DD matrices. This is summarized in Table 9 below. The computations were repeated several times obtaining results with small differences in the values but the same order of magnitude and starting digits.

**Table 9:** Relative volume of embeddable matrices within relevant subsets of $6\times 6$ centrosymmetric Markov matrices. The results were obtained using the hit-and-miss Monte Carlo integration with $10^7$ sample points

Set	Sample points	Embeddable sample points	Rel. vol. of embeddable matrices
$V^{Markov}_6$	$10^8$	1370	0.0000137
$V_{\mathrm{{DLC}}}$	1034607	1362	0.0013164
$V_{\mathrm{{DD}}}$	3048	84	0.0275590

As we have seen in Sect. 3 and 6, we have only considered in detail the embeddability of CS Markov matrices of size $n=4$ and $n=6$. We expect that the proportion of the embeddable CS Markov matrices within the subset of Markov matrices in larger dimension tends to zero as *n* grows larger as indicated by Tables 2, 7, 8, and 9.

These results together with the results obtained for the strand symmetric model (see Table 7) indicate that restricting to homogeneous Markov processes in continuous-time is a very strong restriction because non-embeddable matrices are discarded and their proportion is much larger than that of embeddable matrices. For instance, in the $2\times 2$ case exactly $50\%$ of the matrices are discarded (Ardiyansyah et al. 2021, Table 5), while in the case of $4\times 4$ matrices up to $98.26545\%$ of the matrices are discarded (see Table 7) and in the case of $6\times 6$ matrices the amount of discarded matrices is about $99.99863\%$ as indicated in Table 9. However, when restricting to subsets of Markov matrices which are mathematically more meaningful in biological terms, such as DD or DLC matrices, the proportion of embeddable matrices is much higher so that we are discarding less matrices (e.g. for DD we discard $68.41679\%$ of $4\times 4$ matrices and $97.2441\%$ of $6\times 6$ matrices). This is not to say that it makes no sense to use continuous-time models but to highlight that one should take the above restrictions into consideration when working with these models. Conversely, when working with the whole set of Markov matrices one has to be aware that they might end up considering lots of non-meaningful matrices.
